# The risk factors of glycemic control, blood pressure control, lipid control in Chinese patients with newly diagnosed type 2 diabetes _ A nationwide prospective cohort study

**DOI:** 10.1038/s41598-019-44169-4

**Published:** 2019-05-22

**Authors:** Xiaoling Cai, Dayi Hu, Changyu Pan, Guangwei Li, Juming Lu, Qiuhe Ji, Benli Su, Haoming Tian, Shen Qu, Jianping Weng, Danyi Zhang, Jie Xu, Linong Ji

**Affiliations:** 1 Department of Endocrinology and Metabolism, Peking University People’s Hospital, Beijing, China; 2 Department of Cardiology, Peking University People’s Hospital, Beijing, China; 3 Department of Endocrinology and Metabolism, Chinese People’s Liberation Army General Hospital, Beijing, China; 4 Department of Endocrinology and Metabolism, Fuwai Hospital, Beijing, China; 5 Department of Endocrinology and Metabolism, The Forth Military Medical University Xi Jing Hospital, Xian, China; 6 Department of Endocrinology and Metabolism, The Second Affiliated Hospital Dalian Medical University, Dalian, China; 7 Department of Endocrinology and Metabolism, Sichuan University West China Hospital, Chengdu, China; 8 Department of Endocrinology and Metabolism, Shanghai Tenth People’s Hospital, Shanghai, China; 9 Department of Endocrinology and Metabolism, The Third Affiliated Hospital, Sun Yat-Sen University, Guangzhou, China; 10 VitalStrategic Research Institute, Shanghai, China

**Keywords:** Type 2 diabetes, Epidemiology

## Abstract

Nationwide data on glycemic control, blood pressure (BP) control and lipid control in patients with newly diagnosed type 2 diabetes were vacant in China. The aim of this study was to assess the clinical outcomes for these patients. This is an observational prospective cohort study with 12 months of follow up. Patients with a diagnosis of type 2 diabetes less than 6 months were enrolled. Hemoglobin A1c (HbA1c) levels, BP levels and lipid levels were collected at baseline and the follow-ups. This study was registered at www.clinicaltrials.gov (NCT01525693). A total of 5770 participants from 79 hospitals across six geographic regions of China were recruited. After 12 months of treatment, 68.5% of these patients achieved HbA1c <7.0%; 83.7% reached BP <140/90 mmHg; 48.2% met low density lipoprotein cholesterol (LDL-c) <2.6 mmol/L; and 29.5% of patients reached the combined three therapeutic targets. Compared to those patients with baseline HbA1c <7.0%, patients with baseline HbA1c ≥7.0% had higher failure rate to reach glycemic control (relative risk (RR) = 2.04, p < 0.001), BP control (RR = 1.21, p < 0.001) and LDL-c control (RR = 1.11, p < 0.001). Obese patients had higher possibilities of failure in glucose control (RR = 1.05, p = 0.004), BP control (RR = 1.62, p < 0.001) and lipid control (RR = 1.09, p = 0.001) than patients with normal weight. The active smokers were more likely to fail in glycemic control than non-smokers (RR = 1.06, p = 0.002), and patients with physical activities were less likely to fail in lipid control than patients without exercises (RR = 0.93, p = 0.008). This study outlined the burdens of glycemic control, blood pressure control, lipid control in newly diagnosed type 2 diabetic patients in China, identified gaps in the quality of care and risk-factor control and revealed the factors influencing these gaps.

## Introduction

Type 2 diabetes is characterized by hyperglycemia, insulin resistance, and relative insulin deficiency. It increases the risk of physical disability and life-threatening complications, including microvascular diseases and macrovascular diseases. High-quality evidence from multiple epidemiological studies and large randomized controlled trials demonstrated the relationship between tight glycemic control and a lower risk of diabetes-related complications and mortality^1–4^. Subsequent trials showed that lowering blood pressure and cholesterol levels decreased the incidence of vascular diseases and mortality^5,6^. Moreover, it was suggested intensive glycemic control had a legacy effect which was a beneficial cardiovascular effect^7,8^. Similar legacy findings were also seen with good control of blood pressure^9,10^ and lipid level^11,12^ for diabetic patients. Therefore, early controls of glucose level, blood pressure and blood lipid level are urgent for type 2 diabetic patients^13–15^.

Unfortunately, China has become the country with the largest number of patients with diabetes^16,17^. The prevalence of diabetes increased rapidly to around 11%, with estimated prevalence of 8.1% for newly detected diabetes, which resulted in a huge economic burden for China^18,19^. As recommended by the guidelines^20,21^, effective clinical management in early stage of newly diagnosed type 2 diabetic patients is critically needed to attenuate disease progression and to reduce the complications of diabetes. However, despite good-quality evidence on the legacy benefits of multifactorial risk factor interventions, patients with type 2 diabetes could not reach recommended targets for glycemic level, blood pressure and lipid profile around the world. Although some clinical characteristics and treatment patterns in Chinese type 2 diabetes were reported retrospectively^22–25^, no data has been reported about the controls of multiple risk factors in newly diagnosed type 2 diabetic patients nationwide. Therefore, we designed this prospective, nationwide multicenter, observational cohort study with 12-month follow-up, a study of China Cardiometabolic Registry for newly diagnosed type 2 diabetic patients (CCMR-NEW2D), with the aim of evaluating treatment patterns and their clinical outcomes for newly diagnosed type 2 diabetic patients in China.

## Methods

### Study design and population

This study was a prospective, observational cohort study with 12-month follow up. From June 2012 to February 2014, patients from 81 hospitals (community hospitals (Tier 1), secondary/city level hospitals (Tier 2), and teaching or comprehensive central hospitals (Tier 3)) across six geographic regions of China (North, South, East, Southwest, Northeast, Northwest) were recruited. Participants were enrolled at department of endocrinology and internal medicine clinics. The inclusion criteria were: (1) patients with 20 years’ age or older; (2) patients with confirmed diagnosis of type 2 diabetes according to the World Health Organization criteria, within 6 months before screening; (3) patients who signed the consent form. The exclusion criteria were: (1) patients who were pregnant or breast-feeding or planned to be pregnant within one year; (2) patients who were participating in another clinical trial; (3) patients who were not willing to or not able to return to the same hospital every 3 months for the follow up visits after enrollment; (4) Patients without clear information regarding the medication used. CCMR-NEW2D study was registered at www.clinicaltrials.gov (NCT01525693) on February 3^rd^, 2012 (Supplement eProtocol).

The target of blood glucose control was set as meeting HbA1c <7%. The definitions of hypertension and dyslipidemia were described below. Subjects were classified as hypertension by meeting one of the following three criteria: (1) History of hypertension recorded; (2) Systolic blood pressure ≥130 mmHg or a diastolic blood pressure ≥85 mmHg measured after sitting quietly for at least 5 minutes on at least two successive measurements. There should be 2 minutes interval between the 2 measurements; (3) Anti-hypertensive drug was used by the current visit or would be prescribed at current visit. Two blood pressure treatment targets were set for this analysis: Systolic blood pressure <140 mmHg and diastolic blood pressure <90 mmHg; or Systolic blood pressure <140 mmHg and diastolic blood pressure <80 mmHg. Dyslipidemia was defined as failing to achieve one or more of following lipid levels or receiving lipid regulation therapy: (1) Total Cholesterol (TC) <4.14 mmol/L; (2) High-density Lipoprotein Cholesterol (HDL-c) >1.04 mmol/L; (3) Triglycerides (TG) <1.7 mmol/L; or (4) Low-density Lipoprotein Cholesterol (LDL-c) <2.6 mmol/L. The target of blood lipid management is set as meeting LDL-c <2.6 mmol/L.

Ethical approval was first obtained from the Ethic committees of Peking University People’s Hospital and then was approved by all the participating hospitals. All patients signed the informed consent form before participation. The research methods of the study adhered to the Declaration on Helsinki and all research was reported in accordance with strengthening the reporting of observational studies in epidemiology (STROBE) Statement (Supplement eChecklist).

### Study procedures and data collection

The patients all received routine lifestyle suggestions as diet and exercise by the investigators and also medications prescribed by the investigators. These patients were required to return to the same physician for the follow up visits at 3, 6, 9 and 12 months after the first visit. If the patient was lost of follow up, a structured telephone interview would be performed by the investigator to realize the patient’s condition.

At baseline and the follow-up period, the information as follows should be collected from each patient: (1) Demographics including age, gender, residential region, educational level and social-economic status. (2) Diabetic and family histories. (3) Medical histories. (4) Co-morbidities including hypertension, dyslipidemia, cardiovascular disease, diabetes related complications, cancer. (5) Health behavior (including smoking, drinking (alcohol), were recorded by patient self-reporting. Smoking was defined as current smoker, non-smoking was defined as non-current smoker. Drinking was defined as the frequency of drinking was no less than once a week. Non-drinking was defined as the frequency of drinking was no more than once a week). (6) Physical examinations and laboratory tests including height, body weight, sitting blood pressure, HbA1c, fasting lipid profile. (7) Adverse events, which was defined as any untoward medical occurrence in a patient or clinical investigation subject administered a pharmaceutical product and which did not necessarily have to have a causal relationship with this treatment. The investigators would decide whether the adverse events were associated with the current medications or not according to their clinical experiences. (8) Hypoglycemia was defined as the fasting plasma glucose level below 70 mg/dL according to the American Diabetes Association 2009 Position Statement. The symptoms of hypoglycemia include but not limited to sweating, hunger, trembling, anxiety, confusion, blurred Vision. A simple questionnaire will be used to record patient reported symptoms and severity. (9) Specific information about the hypoglycemic treatments were identified, including diet and physical activities only, use of herbal medicine only, use of anti-diabetic drugs.

All laboratory measurements were performed in the local hospitals where the visits were conducted. For data collection and quality control, all the data were recorded in the approved case report form and entered into a web-based electronic data capture system designed by VitalStrategic Research Institute (VSRI) (Shanghai, China).

### Statistical analysis

Descriptive statistics were used to characterize the data in the study, including calculations of means and standard deviations. The frequency and percentages (based on the non-missing sample size) of observed levels were reported for all categorical measures. Comparisons were statistically analyzed using one-way anova and chi-squared tests. The primary outcome was the overall proportion of patients reaching HbA1c <7.0% at the end of one-year follow-up. Generalized estimating equation (GEE) model was applied for the multiple analyses of primary endpoints to assess relative risks (RRs) and 95% confidence interval (CI). The selections of independent variables were determined by both clinical experiences and factor contribution. GEE model was used to evaluate the influential factors associated with the time to the changes for the hypoglycemic treatment pattern. The models included the three time-dependent variables: hypoglycemic treatment paradigm, study visit and the reason of treatment change; and adjusted for pre-selected baseline characteristics: patient’s blood glucose level, blood pressure and blood lipid level, adequate HbA1c control, gender, age, education, insurance type, family income and health behaviors, *et al*. P value < 0.05 for the two-tailed test was considered as statistically significant. Statistical analyses were conducted using statistical analysis system (SAS) version 9.3 (SAS Institute, Cary, North Carolina, United States of America) (Supplement eProtocol).

### Ethics approval and consent to participate

Ethical approval was first obtained from the Ethic committees of Peking University People’s Hospital and then was approved by all the participating hospitals (Supplemental Table S1 provided a complete list of hospitals and investigators). All patients signed the informed consent form before participation.

### Consent for publication

Consent form for publication was also approved by the Ethic committees of Peking University People’s Hospital and then was approved by all the participating hospitals (Supplemental Table S1 provided a complete list of hospitals and investigators). All patients signed the consent form for publication before participation.

## Results

### Characteristics of newly diagnosed type 2 diabetes patients

Totally 5770 patients from 79 hospitals, across six geographic regions of China, were included in this report. Baseline demographics of newly diagnosed type 2 diabetic patients in China under hypoglycemia treatment patterns are shown in Table 1. The average age of the patients was 55.7 ± 12.6 years and 54.2% were men. The mean body mass index (BMI) of the patients was 25.0 ± 3.4 kg/m^2^. 37.3% of the patients had hypertension and 46.3% of them had dyslipidemia at baseline. 23.6%, 27.3% and 49.0% patients were from tier 1, tier 2 and tier 3 hospitals, respectively.

**Table 1:** Baseline Characteristics of newly diagnosed patients with type 2 diabetes in China.

Characteristics	Total
**All patients, N**	5770
**Age (yr), mean** ± **SD**	55.7 ± 12.6
Age (yr), N (%)	
20–<65 yrs	4408 (76.4%)
≥65 yrs	1362 (23.6%)
Gender, N (%)	
Male	3130 (54.2%)
Female	2640 (45.8%)
Smoking status, N (%)	
None	3902 (67.6%)
Current	1271 (22.0%)
Previous	505 (8.8%)
Passive	92 (1.6%)
Drinking status, N (%)	
None	4860 (84.2%)
Current	619 (10.7%)
Previous	291 (5.0%)
Physical Activities, N (%)	
No exercises	1348 (23.4%)
≤3 times/week	2406 (41.7%)
>3 times/week	2016 (34.9%)
Medicine Compliance, N (%)	
Yes	5278 (91.5%)
No	492 (8.5%)
BMI (kg/m^2^), mean ± SD	25.0 ± 3.4
BMI Category, N (%)	
<24 kg/m^2^	2249 (39.0%)
24–<28 kg/m^2^	2544 (44.1%)
≥28 kg/m^2^	977 (16.9%)
Family history of diabetes: N (%)	
Yes	1628 (28.2)
No	3962 (68.7)
Unknown	180 (3.1)
Family history of cardiovascular disease: N (%)	
No	4429 (76.8)
Yes	1067 (18.5)
Unknown	274 (4.7)
Hypertension, N (%)	2152 (37.3%)
Years of HTN diagnosis, mean ± SD	7.9 ± 8.5
Anti-hypertensive drug use, N (%)	1670 (77.6)
Dyslipidemia, N (%)	2670 (46.3%)
Years of dyslipidemia diagnosis, mean ± SD	1.8 ± 4.0
Lipid-lowering drug use, N (%)	1308 (49.0)
Region	
North	573 (9.9)
South	915 (15.9)
East	782 (13.6)
Southwest	1503 (26.0)
Northeast	856 (14.8)
Northwest	1141 (19.8)
Hospital Tier	
1st tier	1364 (23.6)
2nd tier	1577 (27.3)
3rd tier	2829 (49.0)
Education	
Illiteracy	192 (3.3)
Elementary school	850 (14.7)
Middle school	1658 (28.7)
High school	1502 (26.0)
2–3 Year College	789 (13.7)
4 Year college or over	777 (13.5)
Insurance	
Medical insurance for urban workers	4069 (70.5)
Rural cooperative medical care	782 (13.6)
Public or labor health insurance	345 (6.0)
Commercial Insurance	36 (0.6)
Other medical insurance	121 (2.1)
No insurance	417 (7.2)
Family income	
<500	157 (2.7)
500–2000	1339 (23.2)
2000–5000	2479 (43.0)
>5000	899 (15.6)
Unknown	896 (15.5)
Comorbidities	
Diabetes only	2090 (36.2)
Diabetes + Hypertension	1010 (17.5)
Diabetes + Dyslipidemia	1528 (26.5)
Diabetes + Hypertension + Dyslipidemia	1142 (19.8)
HbA1c % (mmol/mol), mean ± SD	
Total	8.4 ± 2.5 (68 ± 19)
Diet and exercises alone	8.3 ± 2.4 (67 ± 18)
Herbal medicine	7.0 ± 1.6 (53 ± 12)
One OHA, no insulin	7.4 ± 1.9 (57 ± 14)
Two OHAs, no insulin	8.3 ± 2.3 (67 ± 17)
More than two OHAs, no insulin	8.8 ± 2.6 (73 ± 20)
Insulin only, no OHA	9.8 ± 2.7 (84 ± 20)
Insulin + one OHA	10.0 ± 2.7 (86 ± 20)
Insulin + two OHAs	10.2 ± 2.7 (88 ± 20)
Insulin + more than two OHAs	10.5 ± 2.7 (91 ± 20)

### Completion of study visits

As an observational cohort study, the control of study visit window was relatively loose in the process of data cleaning. Under the condition of no influence of statistical analysis, the window period between visits was expanded to ±1 month. Summaries of study visits completion in total and stratified by recruitment characteristics (hospital tier and region) are shown in Table 2.

**Table 2:** Visit completion by hospital tier and regions.

	Baseline N (Row%, Col%)	Visit 1 (3 month) N (Row%, Col%)	Visit 2 (6 month) N (Row%, Col%)	Visit 3 (9 month) N (Row%, Col%)	Visit 4 (12 month) N (Row%, Col%)
Hospital Tier					
1st tier	1364 (23.6, 100.0)	1321 (26.5, 96.8)	1305 (27.1, 95.7)	1268 (27.2, 93.0)	1242 (27.2, 91.1)
2nd tier	1577 (27.3, 100.0)	1416 (28.4, 89.8)	1396 (29.0, 88.5)	1320 (28.3, 83.7)	1286 (28.2, 81.5)
3rd tier	2829 (49.0, 100.0)	2252 (45.1, 79.6)	2116 (43.9, 74.8)	2070 (44.4, 73.2)	2034 (44.6, 71.9)
Regions					
North	573 (9.9, 100.0)	555 (11.1, 96.9)	530 (11.0, 92.5)	501 (10.8, 87.4)	479 (10.5, 83.6)
South	915 (15.9, 100.0)	763 (15.3, 83.4)	705 (14.6, 77.0)	648 (13.9, 70.8)	589 (12.9, 64.4)
East	782 (13.6, 100.0)	663 (13.3, 84.8)	639 (13.3, 81.7)	615 (13.2, 78.6)	603 (13.2, 77.1)
Southwest	1503 (26.0, 100.0)	1238 (24.8, 82.4)	1203 (25.0, 80.0)	1155 (24.8, 76.8)	1145 (25.1, 76.2)
Northeast	856 (14.8, 100.0)	768 (15.4, 89.7)	751 (15.6, 87.7)	738 (15.8, 86.2)	725 (15.9, 84.7)
Northwest	1141 (19.8, 100.0)	1002 (20.1, 87.8)	989 (20.5, 86.7)	1001 (21.5, 87.7)	1021 (22.4, 89.5)
Total	5770 (100.0, 100.0)	4989 (100.0, 86.5)	4817 (100.0, 83.5)	4658 (100.0, 80.7)	4562 (100.0, 79.1)

### Achievements in glycemic control, blood pressure control, lipid control and weight control

**Figure 1:**
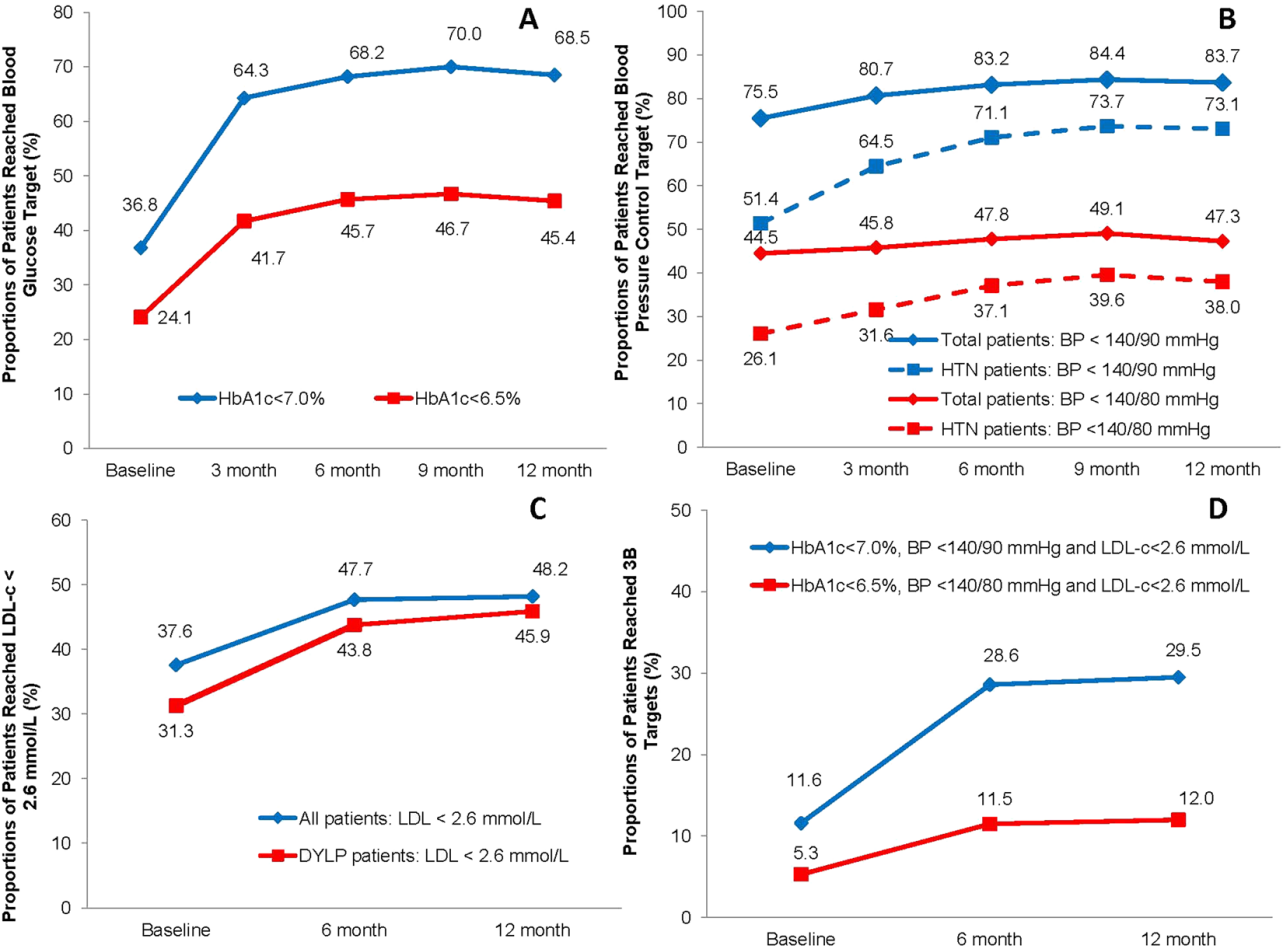
Proportion of patients reaching the target of HbA1c <7.0%, BP <140/90 mmHg, LDL-C <2.6 mmol/L in newly diagnosed type 2 diabetes patients at baseline and during the 12 months of follow-ups. (**A**) Proportion of patients reaching the target of HbA1c <7.0% at baseline and during the 12 months of follow-ups (p < 0.05), proportion of patients reaching the intensive target of HbA1c <6.5% at baseline and during the 12 months of follow-ups (p < 0.05). (**B**) Proportion of patients reaching the target of BP <140/90 mmHg at baseline and during the 12 months of follow-ups in total patients (p < 0.05 for trend) and in patients with hypertension (HTN) (p < 0.05 for trend), proportion of patients reaching the intensive target of BP < 140/80 mmHg at baseline and during the 12 months of follow-ups in total patients (p < 0.05 for trend) and in patients with hypertension (HTN) (p < 0.05 for trend). (**C**) Proportion of patients reaching the target of LDL-C <2.6 mmol/L at baseline and during the 12 months of follow-ups in total patients (p < 0.05 for trend) and in patients with dyslipidemia (DYLP) (p < 0.05 for trend). (**D**) Proportion of patients reaching the combined three therapeutic targets (HbA1c <7.0%, BP <140/90 mmHg and LDL-C <2.6 mmol/L) at baseline and during the 12 months of follow-ups (p < 0.05 for trend); proportion of patients reaching the combined three therapeutic targets (HbA1c <6.5%, BP <140/80 mmHg and LDL-C <2.6 mmol/L) at baseline and during the 12 months of follow-ups (p < 0.05 for trend).

**Figure 2:**
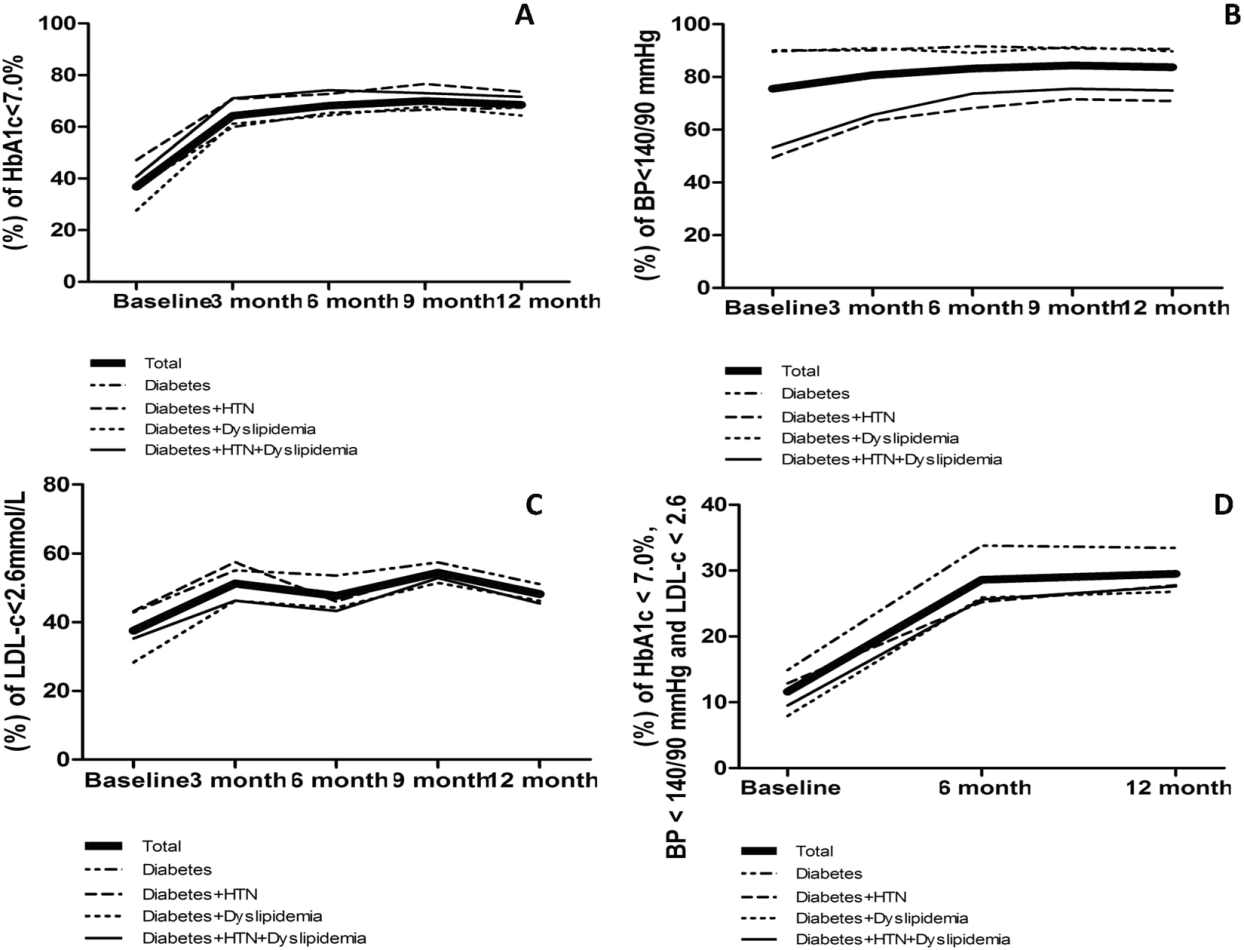
Proportion of patients reaching the target of HbA1c <7.0%, BP <140/90 mmHg, LDL-C <2.6 mmol/L in newly diagnosed type 2 diabetes patients at baseline and during the 12 months of follow-ups categorized by comorbidities. (**A**) Proportion of patients reaching the target of HbA1c <7.0% at baseline and during the 12 months of follow-ups in total patients (p < 0.05 for trend), in patients with diabetes only (p < 0.05 for trend), in patients with diabetes and hypertension (p < 0.05 for trend), in patients with diabetes and dyslipidemia (p < 0.05 for trend), in patients with diabetes, hypertension and dyslipidemia (p < 0.05 for trend). (**B**) Proportion of patients reaching the target of BP <140/90 mmHg at baseline and during the 12 months of follow-ups in total patients (p < 0.05 for trend), in patients with diabetes only (p < 0.05 for trend), in patients with diabetes and hypertension (p < 0.05 for trend), in patients with diabetes and dyslipidemia (p < 0.05 for trend), in patients with diabetes, hypertension and dyslipidemia (p < 0.05 for trend). (**C**) Proportion of patients reaching the target of LDL-C <2.6 mmol/L at baseline and during the 12 months of follow-ups in total patients (p < 0.05 for trend), in patients with diabetes only (p < 0.05 for trend), in patients with diabetes and hypertension (p < 0.05 for trend), in patients with diabetes and dyslipidemia (p < 0.05 for trend), in patients with diabetes, hypertension and dyslipidemia (p < 0.05 for trend). (**D**) Proportion of patients reaching the three targets of HbA1c <7.0%, BP <140/90 mmHg and LDL-C <2.6 mmol/L altogether at baseline and during the 12 months of follow-ups in total patients (p < 0.05 for trend), in patients with diabetes only (p < 0.05 for trend), in patients with diabetes and hypertension (p < 0.05 for trend), in patients with diabetes and dyslipidemia (p < 0.05 for trend), in patients with diabetes, hypertension and dyslipidemia (p < 0.05 for trend).

**Figure 3:**
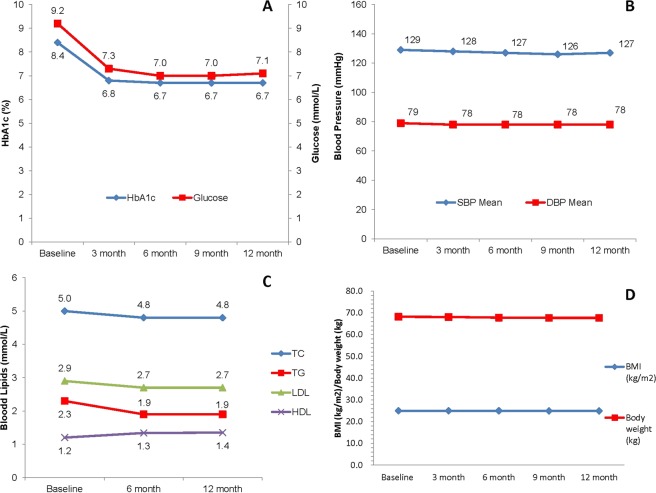
Changes in HbA1c and glucose level, SBP and DBP, lipid profiles, body weight and BMI in newly diagnosed type 2 diabetic patients within 12 months of follow-ups. (**A**) Changes in HbA1c (p < 0.05 for trend) and fasting glucose level (p < 0.05 for trend) within 12 months of follow-ups. (**B**) Changes in SBP (p < 0.05 for trend) and DBP (p < 0.05 for trend) within 12 months of follow-ups. (**C**) Changes in TC (p < 0.05 for trend), TG (p < 0.05 for trend), LDL-c (p < 0.05 for trend) and HDL-c (p < 0.05 for trend) within 12 months of follow-ups. (**D**) Changes in body weight (p < 0.05 for trend) and BMI (p < 0.05 for trend) within 12 months of follow-ups.

Overall, the mean HbA1c of the total population was 8.4 ± 2.5% at the time of diagnosis. 36.8% of the patients reached the glycemic control target of HbA1c <7.0%. After 12-month treatment, the mean HbA1c decreased to 6.7 ± 1.2% and 68.5% of the patients reached HbA1c <7.0%. Moreover, nearly one fourth (24.1%) of patients reached the stricter target goal of HbA1c < 6.5% at baseline, and by the end of 12-month follow-up, 45.4% of them reached HbA1c <6.5% (Figs 1A, 2A and 3A). There was also a significantly linear trend of reaching adequate glycemic control (P < 0.0001).

The mean systolic blood pressure slightly decreased to 127 ± 12 mmHg after 12 months (P < 0.0001) and the mean diastolic blood pressure decreased to 78 ± 7 mmHg after 12 months (P < 0.001). Overall, 75.5% of the total population reached the recommended target of BP <140/90 mmHg at baseline, and 83.7% of them reached target BP after 12 months (Figs 1B, 2B and 3B). Among 2152 patients with hypertension at baseline, 51.4% of them reached target BP at baseline and 73.1% reached target BP after 12 months (P < 0.0001).

The mean levels of TC, TG, and LDL-c decreased significantly after 12 months (P < 0.0001), and the mean level of HDL-c increased significantly after 12 months (P < 0.0001). Overall, the proportion of patients who achieved the recommended blood lipids target (LDL-c <2.6 mmol/L) was 37.6% at baseline and increased to 48.2% after 12 months (P < 0.0001). Among 2670 patients who had been confirmed dyslipidemia at baseline, the proportion of patients who achieved LDL-c target was 31.3% at baseline and increased to 45.9% after 12 months (Figs 1C, 2C and 3C).

The proportions of patients, achieving the combined therapeutic targets of HbA1c <7.0%, BP <140/90 mmHg and LDL-c <2.6 mmol/L, doubled from 11.6% to 29.5% after one-year follow-up (P < 0.0001). And the proportion increased from 5.3% to 12.0% if using the stricter control target of HbA1c <6.5%, BP <140/80 mmHg and LDL-c <2.6 mmol/L (P < 0.0001) (Figs 1D and 2D).

The mean BMI of the overall patients was 25.0 ± 3.4 kg/m^2^ and the mean body weight was 68.2 ± 12.1 kg at baseline, with 44.1% overweight (BMI: 24–<28 kg/m^2^) and 16.9% obese (BMI ≥28 kg/m^2^). The mean BMI slightly decreased to 24.9 kg/m^2^ after one-year (P < 0.05), with 58.5% overweight or obese (Fig. 3D).

Proportions of patients with adequate control of three therapeutic targets, regarding different characteristics including patient demography, health behavior, and hospital tier, were described in Table 3.

**Table 3:** Proportion of T2D patients reached HbA1c <7.0%, BP <140/90 mmHg and LDL-c <2.6 mmol/L by Patient Characteristics.

Characteristics	HbA1c <7.0%		BP <140/90 mmHg		LDL-c <2.6 mmol/L		All the three targets reached	
	Baseline	12 month	Baseline	12 month	Baseline	12 month	Baseline	12 month
**Total**	2096 (36.8)	3073 (68.5)	4358 (75.5)	3819 (83.7)	2139 (37.6)	2113 (48.2)	655 (11.6)	1283 (29.5)
Gender								
Male	988 (32.0)	1558 (65.9)	2363 (75.5)	2023 (83.7)	1203 (39.1)	1152 (49.7)	320 (10.5)	677 (29.4)
Female	1108 (42.6)	1515 (71.5)	1995 (75.6)	1796 (83.7)	936 (35.8)	961 (46.6)	335 (13.0)	606 (29.6)
Age (year)								
20–<65	1458 (33.5)	2288 (68.0)	3436 (77.9)	2958 (86.2)	1608 (37.0)	1599 (48.5)	476 (11.1)	996 (30.4)
>=65	638 (47.8)	785 (70.2)	922 (67.7)	861 (76.1)	531 (39.4)	514 (47.5)	179 (13.5)	287 (26.7)
Smoking								
None	1569 (40.9)	2142 (70.0)	2960 (75.9)	2595 (83.6)	1426 (37.0)	1421 (47.7)	482 (12.7)	867 (29.3)
Current	331 (26.2)	601 (62.2)	967 (76.1)	835 (84.2)	484 (38.8)	462 (48.6)	115 (9.3)	264 (28.0)
Previous	157 (31.6)	265 (71.0)	364 (72.1)	324 (84.8)	203 (40.8)	195 (53.0)	50 (10.1)	127 (34.8)
Passive	39 (42.9)	65 (78.3)	67 (72.8)	65 (78.3)	26 (28.9)	35 (42.2)	8 (8.9)	25 (30.1)
Drinking								
None	1852 (38.6)	2626 (69.1)	3700 (76.0)	3246 (84.0)	1790 (37.3)	1777 (47.9)	576 (12.1)	1087 (29.5)
Current	174 (28.2)	302 (64.0)	450 (72.6)	401 (83.4)	239 (39.2)	225 (48.4)	59 (9.7)	129 (27.9)
Previous	70 (25.0)	145 (69.7)	208 (73.5)	172 (80.0)	110 (39.3)	111 (53.9)	20 (7.2)	67 (33.0)
Physical Activities								
No exercises	405 (30.3)	660 (64.5)	996 (73.9)	870 (82.5)	474 (35.8)	460 (46.3)	117 (8.9)	266 (27.2)
Occasional exercises	841 (35.5)	1279 (68.3)	1829 (76.0)	1590 (83.9)	929 (39.0)	892 (48.8)	275 (11.7)	534 (29.4)
Regular exercises	850 (42.8)	1134 (71.4)	1533 (76.0)	1359 (84.3)	736 (37.1)	761 (48.8)	263 (13.3)	483 (31.1)
Medication Compliance								
Yes	1844 (35.5)	2754 (68.1)	4005 (75.9)	3473 (84.3)	1980 (38.1)	1911 (48.5)	597 (11.6)	1166 (29.8)
No	252 (51.6)	319 (72.7)	353 (71.7)	346 (78.5)	159 (32.4)	202 (46.0)	58 (11.9)	117 (26.7)
BMI (kg/m^2^)								
<24.0	790 (35.7)	1308 (70.3)	1803 (80.2)	1662 (87.9)	875 (39.5)	920 (51.0)	271 (12.4)	602 (33.6)
24.0–<28.0	978 (39.1)	1339 (69.2)	1905 (74.9)	1642 (83.5)	940 (37.4)	892 (47.0)	301 (12.1)	543 (28.9)
≥28.0	328 (33.7)	426 (62.0)	650 (66.5)	515 (73.2)	324 (33.7)	301 (44.3)	83 (8.6)	138 (20.5)
Patient Category								
Diabetes	752 (36.6)	1091 (67.4)	1880 (90.0)	1490 (90.5)	882 (42.9)	808 (51.1)	303 (14.9)	524 (33.4)
Diabetes + HTN	471 (47.1)	582 (73.5)	500 (49.4)	567 (70.9)	432 (43.2)	375 (48.5)	128 (12.9)	214 (27.8)
Diabetes + Dyslipidemia	415 (27.6)	752 (64.4)	1371 (89.6)	1071 (89.7)	428 (28.3)	528 (46.2)	118 (7.9)	304 (26.8)
Diabetes + HTN + Dyslipidemia	458 (40.7)	648 (71.6)	607 (53.2)	691 (74.9)	397 (35.3)	402 (45.5)	106 (9.5)	241 (27.6)
Family Diabetics History								
No	1493 (38.2)	2094 (68.8)	2972 (75.0)	2583 (83.2)	1503 (38.5)	1446 (48.7)	459 (11.9)	873 (29.7)
Yes	522 (32.5)	865 (67.0)	1252 (76.9)	1111 (84.8)	575 (35.8)	592 (46.6)	176 (11.1)	360 (28.5)
Unknown	81 (46.0)	114 (77.0)	134 (74.4)	125 (83.9)	61 (34.5)	75 (53.6)	20 (11.4)	50 (35.7)
Family CVD History								
No	1572 (36.0)	2276 (67.1)	3379 (76.3)	2889 (83.6)	1663 (38.1)	1598 (48.2)	494 (11.4)	939 (28.6)
Yes	410 (39.0)	636 (73.3)	769 (72.1)	737 (83.7)	390 (36.9)	409 (47.9)	140 (13.4)	281 (33.1)
Unknown	114 (41.9)	161 (71.9)	210 (76.6)	193 (85.8)	86 (31.5)	106 (50.0)	21 (7.7)	63 (29.7)
Region								
North	242 (42.9)	374 (78.1)	489 (85.3)	437 (91.2)	205 (36.0)	197 (41.3)	95 (16.9)	151 (31.7)
South	426 (47.0)	437 (75.0)	688 (75.2)	487 (82.7)	314 (34.5)	221 (38.2)	123 (13.6)	152 (26.3)
East	283 (36.7)	414 (69.1)	609 (77.9)	503 (83.4)	292 (38.3)	306 (51.3)	102 (13.5)	188 (31.6)
Southwest	478 (32.8)	756 (69.7)	1148 (76.4)	960 (83.8)	620 (42.2)	563 (55.7)	154 (10.7)	334 (33.7)
Northeast	250 (29.4)	434 (60.1)	584 (68.2)	621 (85.7)	256 (30.3)	344 (48.0)	63 (7.5)	180 (25.2)
Northwest	417 (36.6)	658 (64.8)	840 (73.6)	811 (79.4)	452 (39.8)	482 (48.2)	118 (10.4)	278 (28.0)
Hospital Classification								
1st tier	893 (65.7)	908 (73.3)	1005 (73.7)	980 (78.9)	494 (36.3)	606 (49.0)	270 (19.9)	371 (30.1)
2nd tier	457 (29.2)	842 (65.6)	1131 (71.7)	1109 (86.2)	559 (35.8)	512 (40.3)	133 (8.5)	311 (24.5)
3rd tier	746 (27.0)	1323 (67.4)	2222 (78.5)	1730 (85.1)	1086 (39.2)	995 (53.1)	252 (9.3)	601 (32.6)
Hypoglycemic treatment pattern								
None	931 (37.3)	749 (73.8)	/	/	/	/	259 (10.5)	284 (29.1)
Chinese Herbs only	33 (68.8)	34 (69.4)	/	/	/	/	7 (14.6)	11 (22.4)
One OHD	666 (51.5)	1121 (74.3)	/	/	/	/	223 (17.3)	466 (31.6)
Two OHD	258 (35.3)	600 (67.3)	/	/	/	/	95 (13.1)	257 (29.6)
Over Two OHD	39 (32.5)	82 (59.9)	/	/	/	/	13 (10.8)	33 (25.0)
Insulin Alone	103 (18.7)	259 (57.6)	/	/	/	/	37 (6.9)	116 (26.7)
Insulin + One OHD	47 (15.1)	166 (55.1)	/	/	/	/	15 (4.9)	87 (30.0)
Insulin + Two OHD	15 (12.6)	53 (47.7)	/	/	/	/	4 (3.4)	26 (24.8)
Insulin + Over Two OHD	4 (16.7)	9 (45.0)	/	/	/	/	2 (8.7)	3 (15.8)

### Associated factors with the failure of achieving glycemic control, BP control and LDL-c control

After 12 months treatment in newly diagnosed patients with type 2 diabetes, 31.5% of them still failed to achieve HbA1c target, with 16.3% not reaching the target of BP control and 51.8% failing to achieve the goal of LDL-c control. The proportion of patients with adequate control of three therapeutic targets was 70.5%. The associated factors with failure to achieve glycemic control, BP control and lipid control were shown in Table 4.

**Table 4:** Associations of Baseline and Time-varying Risk Factors on the failure of HbA1c, blood pressure and LDL-c controls.

Characteristics	HbA1c ≥7.0%			BP ≥140/90 mmHg			LDL-c ≥2.6 mmol/L		
	RR (95%CI)	P value	Global P value	RR (95% CI)	P value	Global P value	RR (95% CI)	P value	Global P value
Medication groups:									
None or herbal medicine only	Ref.		<0.0001	Ref.		<0.0001	Ref.		<0.0001
One OHD	0.93 (0.90–0.96)	<0.0001		0.83 (0.76–0.90)	<0.0001		0.92 (0.89–0.96)	0.0002	
Two more OHD	0.98 (0.90–1.05)	0.5284		0.78 (0.70–0.86)	<0.0001		0.87 (0.83–0.91)	<0.0001	
Insulin Alone	1.06 (1.01–1.11)	0.0194		0.82 (0.72–0.94)	0.0037		0.90 (0.84–0.95)	0.0007	
Insulin + One OHD	1.09 (1.03–1.15)	0.0034		0.76 (0.65–0.89)	0.0008		0.83 (0.77–0.90)	<0.0001	
Insulin + ≥ Two OHD	1.14 (1.05–1.23)	0.0050		0.83 (0.65–1.05)	0.1187		0.78 (0.69–0.89)	0.0002	
Patient Category:									
Diabetes only	Ref.		<0.0001	Ref.		<0.0001	Ref.		<0.0001
Diabetes + Hypertension	0.93 (0.90–0.96)	<0.0001		3.67 (3.26–4.14)	<0.0001		1.04 (0.98–1.10)	0.2417	
Diabetes + Dyslipidemia	0.99 (0.96–1.02)	0.6538		1.09 (0.94–1.27)	0.2689		1.18 (1.13–1.24)	<0.0001	
Diabetes + Hypertension + Dyslipidemia	0.95 (0.91–0.98)	0.0010		3.37 (2.99–3.80)	<0.0001		1.12 (1.06–1.18)	<0.0001	
Baseline HbA1c level:									
<7.0%	Ref.		<0.0001	Ref.		<0.0001	Ref.		<0.0001
>=7.0%	2.04 (1.98–2.10)	<0.0001		1.21 (1.13–1.30)	<0.0001		1.11 (1.07–1.15)	<0.0001	
BMI groups:									
<24 kg/m^2^	Ref.		0.02	Ref.		<0.0001	Ref.		0.0040
24–28 kg/m^2^	1.01 (0.99–1.04)	0.2124		1.18 (1.08–1.28)	0.0003		1.04 (1.00–1.08)	0.0579	
>=28 kg/m^2^	1.05 (1.02–1.08)	0.0044		1.62 (1.46–1.79)	<0.0001		1.09 (1.04–1.15)	0.0009	
Gender:									
Male	Ref.		0.21	Ref.		0.0055	Ref.		0.0061
Female	0.98 (0.96–1.01)	0.2156		0.87 (0.80–0.96)	0.0047		1.07 (1.02–1.12)	0.0064	
Age groups:									
<65 years	Ref.		0.006	Ref.		<0.0001	Ref.		0.0744
>=65 years	1.03 (1.01–1.06)	0.0067		1.27 (1.16–1.39)	<0.0001		1.04 (1.00–1.09)	0.0719	
Smoking									
None	Ref.		0.004	Ref.		0.3268	Ref.		0.3430
Current	1.06 (1.02–1.09)	0.0021		0.94 (0.84–1.05)	0.2861		1.02 (0.96–1.07)	0.5952	
Previous	0.99 (0.94–1.04)	0.7341		0.88 (0.76–1.03)	0.1110		0.99 (0.91–1.07)	0.7688	
Passive	0.94 (0.86–1.02)	0.1555		1.09 (0.87–1.36)	0.4649		1.12 (0.99–1.26)	0.0761	
Drinking									
None	Ref.		0.07	Ref.		0.2766	Ref.		0.2335
Current	0.97 (0.92–1.02)	0.1892		1.08 (0.95–1.23)	0.2350		0.97 (0.91–1.04)	0.3779	
Previous	0.93 (0.86–1.00)	0.0370		1.13 (0.94–1.35)	0.1832		0.92 (0.84–1.02)	0.1235	
Physical Activities									
No exercises	Ref.		0.71	Ref.		0.7118	Ref.		0.0078
<3 times/week	0.99 (0.95–1.02)	0.4248		0.97 (0.88–1.06)	0.4677		0.93 (0.88–0.97)	0.0018	
>=3 times/week	0.99 (0.95–1.02)	0.4686		0.96 (0.87–1.06)	0.4509		0.94 (0.90–0.99)	0.0204	
Site area									
North	Ref.		<0.0001	Ref.		<0.0001	Ref.		<0.0001
South	1.04 (1.01–1.08)	0.0258		1.90 (1.57–2.30)	<0.0001		1.07 (1.00–1.15)	0.0603	
East	1.08 (1.04–1.12)	0.0001		1.71 (1.41–2.08)	<0.0001		0.98 (0.90–1.06)	0.5534	
Southwest	1.08 (1.04–1.11)	<0.0001		2.03 (1.69–2.46)	<0.0001		0.84 (0.78–0.90)	<0.0001	
Northeast	1.12 (1.08–1.16)	<0.0001		2.47 (2.03–3.01)	<0.0001		1.01 (0.94–1.09)	0.7283	
Northwest	1.14 (1.11–1.17)	<0.0001		2.13 (1.78–2.56)	<0.0001		0.95 (0.88–1.02)	0.1279	
Hospital tier									
1st tier	Ref.		<0.0001	Ref.		0.0220	Ref.		0.0003
2nd tier	0.98 (0.95–1.01)	0.2882		0.89 (0.79–0.99)	0.0377		1.08 (1.03–1.14)	0.0024	
3rd tier	0.90 (0.87–0.94)	<0.0001		0.87 (0.79–0.96)	0.0058		0.99 (0.94–1.05)	0.7810	

In terms of baseline characteristics, compared to those patients with baseline HbA1c <7.0%, patients with baseline HbA1c ≥7.0% had higher possibilities of failing to reach adequate glycemic control (RR = 2.04; P < 0.0001), BP control (RR = 1.21; P < 0.0001) and LDL-c control (RR = 1.11; P < 0.0001) during the follow-up visits (Table 3). This multivariate model also suggested that the older patients (≥65 years) tended to have slightly higher possibilities of failure in glycemic control (RR = 1.03, P = 0.005) and BP control (RR = 1.27, P < 0.0001). Patients with obesity were more likely to fail in glucose control (RR = 1.05; P = 0.0044), BP control (RR = 1.62; P < 0.0001) and LDL-c control (RR = 1.09; P = 0.0009).

In terms of health behavior, the active smokers were more likely to fail in glycemic control than non-smokers (RR = 1.06; P = 0.0021), and patients with physical activities were less likely to fail in lipid control than patients without exercises (RR = 0.93; P = 0.0078). However, the effects of other health behaviors (including drinking, physical activities, and medication compliance) on glycemic controls did not reach statistical significance.

When categorized by comorbidities, it was indicated that compared to diabetic patients without hypertension and dyslipidemia, diabetic patients with hypertension, or diabetic patients with both hypertension and dyslipidemia had lower possibilities of failure in glycemic control (RR = 0.93; *P* < 0.0001 and RR = 0.94; *P* = 0.0008, respectively), but had higher possibilities of failure in BP control (RR = 3.67; *P* < 0.0001 and RR = 3.37; *P* < 0.0001, respectively). Moreover, compared to diabetic patients without hypertension and dyslipidemia, diabetic patients with dyslipidemia or with both hypertension and dyslipidemia, tended to have a higher possibility of failure in LDL-c control (RR = 1.18; *P* < 0.0001 and RR = 1.12; *P* < 0.0001, respectively).

Stratified by treatment patterns, compared to the patients without any hypoglycemic medications or on herbals only, newly diagnosed patients with one oral hypoglycemic agent (OHA) were less likely to fail in glycemic control (RR = 0.93; *P* < 0.001), BP control (RR = 0.83; *P* < 0.001) and LDL-c control (RR = 0.92; *P* < 0.001). Patients with insulin alone (RR = 1.07; *P* = 0.01), insulin plus one OHA (RR = 1.10; *P* = 0.003) and insulin plus two OHAs or more (RR = 1.15; *P* = 0.005), were more likely to fail in glycemic control but were less likely to fail in BP control or LDL-c control (Table 4).

Finally, when categorized by the distributions of regions and hospitals, it was suggested that patients from Tier 3 hospitals had lower possibilities of failure in glycemic control (RR = 0.90; P < 0.0001) and BP control (RR = 0.87; P = 0.0058) than Tier 1 hospitals. The glycemic control and BP control differences also existed across six geographic recruitment regions in China (P < 0.0001).

### Adverse events, serious adverse events and hypoglycemia

A total of 283 adverse events were reported in 263 patients at baseline and during one-year follow-up: 104 cases of gastrointestinal reactions (36.8%), 67 cases of liver or renal dysfunction (23.7%), 24 cases of weight gain (8.5%), 12 cases of edema (4.2%), 9 cases of allergic reaction (3.2%) and other discomforts were reported in 74 cases (40.4%). Among these adverse event cases, 83.3% were initially occurred and 16.7% were recurred.

**Figure 4:**
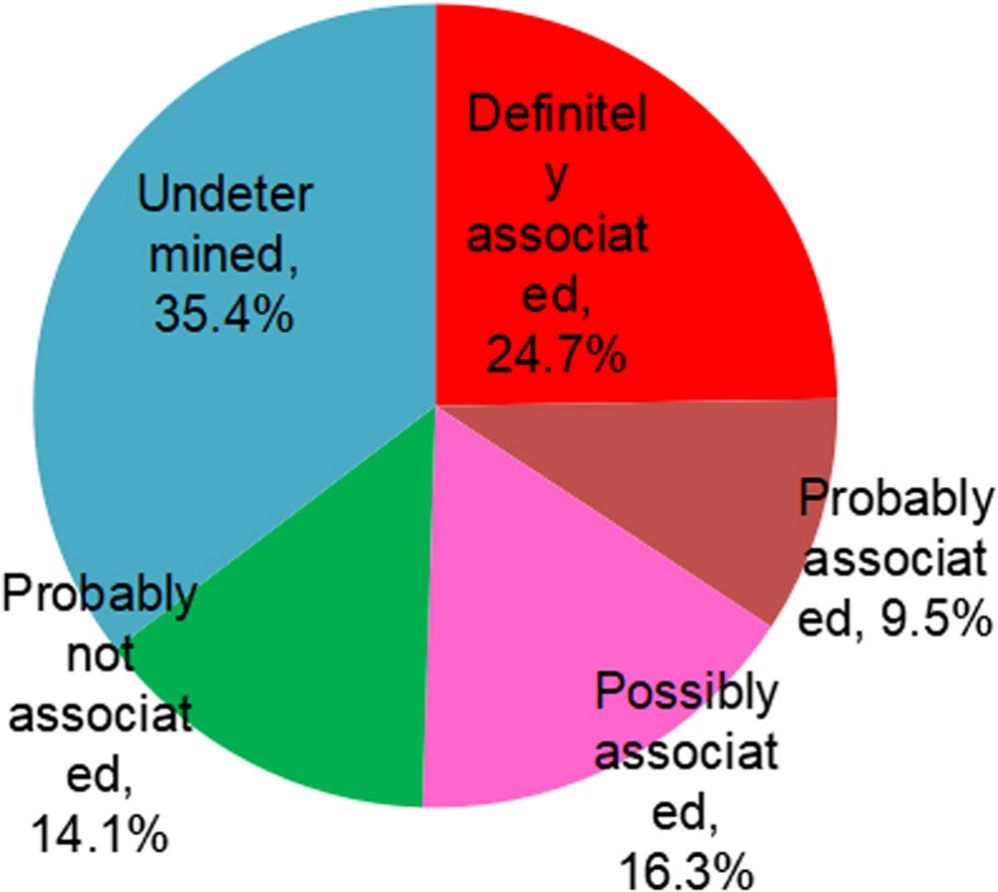
Association between adverse event and current medication.

**Figure 5:**
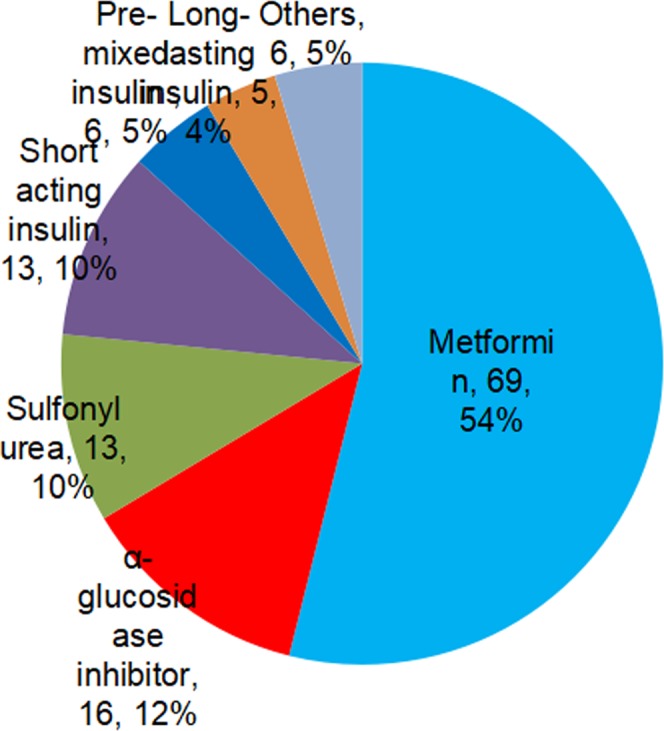
Proportion of hypoglycemic medications identified in reported adverse events.

Of the 283 recorded adverse events, 143 adverse events were definitely (24.7%), probably (9.5%) or possibly (16.3%) associated with the current medication, and nearly half of the adverse events (49.5%) were considered not associated or undetermined with the current medications (Fig. 4). Furthermore, 128 adverse events were considered to be associated with the current hypoglycemic medications. The proportions of the identified hypoglycemic medications are shown in Fig. 5.

A total of 403 severe adverse events were recorded in this study. Among these severe adverse events, only two patients developed serious adverse events (SAE) which caused prolonged hospitalizations due to current medications. The first SAE was reported as liver pain, which required prolonged hospitalization, was evaluated as possibly associated with metformin sustained released tablets. This patient was also on sulfonylurea. The patient recovered after discontinuation of the suspected medication. The other SAE was reported as allergic reaction from glipizide which required prolonged hospitalization. The patient was recovered after discontinuation the suspected medication.

Approximately 8.2% patients had the experience of hypoglycemic episode (HE) prior to baseline. The proportions of patients developing one or more than one hypoglycemia episode since previous study visit were 5.0%, 3.2%, 2.4% and 2.7% at 3, 6, 9 and 12 months, respectively. The majority of patients developed mild hypoglycemia episodes and the rate of HEs decreased during the study period (*P* < 0.0001). The detailed hypoglycemia episodes and severity are shown in Table 5.

**Table 5:** Hypoglycemia Events and Severity.

	Baseline	3 months	6 months	9 months	12 months
Mild HEs	395 (6.85)	231 (4.63)	136 (2.83)	120 (2.20)	111 (2.44)
Moderate HEs	22 (0.38)	17 (0.34)	14 (0.29)	4 (0.09)	7 (0.15)
Severe HEs	8 (0.14)	0 (0.00)	3 (0.06)	1 (0.02)	2 (0.04)
Very severe HEs	7 (0.12)	5 (0.10)	4 (0.08)	1 (0.02)	2 (0.04)

## Discussion

CCMR-NEW2D study was a pioneering large-scale prospective cohort study, to investigate clinical outcomes in newly diagnosed type 2 diabetic patients in China. Overall, this longitudinal prospective cohort study provided the first nationwide data of glycemic control, blood pressure control, lipid control as well as body weight control in newly diagnosed type 2 diabetic patients in China. Achievements in multiple risk factors control showed that 68.5% of these patients met HbA1c <7%, 83.7% of them achieved BP <140/90 mmHg, and 48.2% reached LDL-c <2.6 mmol/L, with only 29.5% of them achieving the three combined therapeutic targets after one-year treatment. Compared with other Chinese retrospective surveys but not newly diagnosed patients, which reported that 39.7% met the glycemic target in population-based study^18^ and 31.78% to 47.7% reached glycemic control in hospital-based studies^22,24,25^, patients with newly diagnosed diabetes in this national study achieved better control after 12 months of follow up.

However, according to this study, after 12 months of treatment, 31.5% of patients still failed to achieve the HbA1c target, 16.3% of them could not reach the BP target and 51.8% failed to achieve the LDL-c goal, with 58.5% being overweight or obese. Overall, 70.5% of these patients could not meet the three combined therapeutic goals, suggesting that there were still heavy burdens in multiple risk factors control in Chinese newly diagnosed diabetic patients. This has profound implications since that the incidence and prevalence of diabetes have been demonstrated to increase rapidly in China^17–19^, and the number of newly diagnosed patients will grow quite a lot accordingly. However, without a well control of multiple risk factors in newly diagnosed diabetic patients, it could be estimated that higher rates of morbidity and mortality would be present early due to severe microvascular and macrovascular complications.

It was well recognized that management of glucose, blood pressure and dyslipidemia could benefit diabetic people in the improvement of microvascular and macrovascular complications. Legacy effect indicating the prolonged benefits of multifactorial risk factor interventions^26,27^ further urged the importance of early risk factor control in primary prevention of cardiovascular disease in type 2 diabetic patients. However, despite of these, it has been estimated that there were still substantial uncontrolled diabetic patients around the world. The national data over the period of 2007–2010 in the United States illustrated that among the study participants, 20.9% failed to achieve glycemic control, 72.2% did not achieve BP control, and 56.8% failed to achieve lipid control^13^. Surveys from Germany during 2008–2011^28^ and in 2014^29^ outlined similar figures as those in United States and indicated that only 11.4% of patients met the combination goals. Our findings were similar with the results from the United States and Germany, except that the proportions of patients achieved the BP target were higher in these newly diagnosed diabetic patients, which revealed that patients with type 2 diabetes around the world need to improve their multi-cardiometabolic risk factor controls of diabetes.

According to the analysis of the possible associated factors with the sub-optimal controls of glucose and other cardiovascular risk factors, we noted that patients with higher levels of baseline HbA1c were less likely to reach adequate glycemic control, BP control and LDL-c control, which urged to consider the importance of earlier diagnosis of diabetes in China for better controls of risk factors. In terms of patient characteristics, it was found that the older adults were less likely to meet the targets of glycemic control and BP control than younger adults with newly diagnosed diabetes, which was opposite to the previous studies in general population with diabetes in the United States but not newly diagnosed individuals^13^, suggesting that newly diagnosed diabetic patients with older age might require more care at the time of diagnosis. Newly diagnosed diabetic patients with comorbidities were less likely to attain the targets of BP control and LDL-c control but more likely to achieve the target of glycemic control, indicating that patients with comorbidities need further attention on multiple controls of cardiovascular risk factors.

Interestingly, we found that compared to patients without any hypoglycemic medications, newly diagnosed patients with one OHA were more likely to attain the multiple targets, which was quite different from the previous findings in general diabetic patients indicating that individuals not taking any diabetes medications were more likely to achieve glycemic control^13,30^. Reasons accounting for the difference might be that participants in our data were all newly diagnosed diabetic patients. Therefore, duration of diabetes shall not be considered as a factor associated with the attainment of target. Moreover, newly diagnosed patients without hypoglycemic medications might be associated with poor compliance to medication, for that they had higher levels of HbA1c at baseline than patients with one OHA, but without any hypoglycemic drugs. Additionally, we observed that patients receiving insulin treatment were less likely to achieve the goal of glycemic control, in accordance with the United States data that only 30% of patients receiving insulin achieved an HbA1c <7.0%^30^, with an explanation that glycemic control is more difficult in individuals with more severe ß-cell loss.

Furthermore, in terms of health behavior, we revealed that patients with obesity were more likely to fail in the control of multiple risk factors than patients with normal weight. And active smokers were less likely to meet the glycemic control than non-smokers, while patients without exercise tended to be less likely to achieve the lipid control than patients with exercise. Underlying reasons might be that individuals may lack self-management skills or the resources necessary for adherence. Although lifestyle behaviors, such as balance diet, physical activity, smoke cessation, need to be sustained in patients with diabetes, it was expected to be always challenging for them to improve and maintain the healthy lifestyles^31^.

We also observed that patients from higher tier hospitals had lower failure rate in glycemic control and BP control than lower tier hospitals, and the differences of glycemic and BP control also existed across six geographic recruitment regions in China, some of which has been shown previously^24,32^. It was indicated that level of hospital, physician professionals and attentiveness in different hospitals and regions, and access to health care in different regions and hospitals might be the possible associated factors with the control of cardiovascular risk factors. Other reasons might be that achieving guideline recommendations may be biologically unattainable for some patients due to severity of disease, other complications, cost, and patient preferences^20,21^. Therefore, individual goals are increasingly being tailored on the basis of individual factors. Side effects of drugs may also limit their use and, therefore, lead to failure to achieve treatment goals.

Gaps were observed between real-world diabetes management and the recommendations for the treatment targets in this study with newly diagnosed diabetic patients. As previously indicated^33–35^, there were lots of challenges for implementing evidence into practice in relation to diabetes prevention, treatment and management across the world. In the achievement of recommended targets, in the adherence to guidelines, and in the adherence to recommended treatments, we should search for evidence-based, patient-centered solutions for those newly diagnosed patients.

As an observational cohort study, there are some limitations. Firstly, no further correction was done for the value of HbA1c in CCMR-NEW2D study. The results of lab test from variable hospitals were accepted, taking real-world evidence and study cost into consideration. Fortunately, in recent years, a series of industry standards have been implemented, thus the reference difference among individual labs become small. Secondly, as an observational study with 12 months follow up, this duration of follow up is not enough to give us comprehensive answers about the clinical outcomes such as macrovascular and microvascular complications associated with the hypoglycemic treatment patterns. Thirdly, the influence of more factors such as eating habits, patient’s professions and the safety of long-term use of medicines was not collected. Thus, multiple-year longitudinal cohort study will be further needed. Fourthly, the HbA1c target for older patients is still controversial. However, as this study is an observational cohort study, with the aim to evaluate clinical outcomes and glycemic control in the real-world in China, as the recommendation from the 2010 Chinese Guideline for Diabetes Prevention and Treatment did not set the HbA1c target for older patients, we did not stratify the target of glucose control for different ages in this study. Moreover, as a real-world observational study, selection bias could not be fully avoided.

## Conclusions

In summary, achievements in multiple risk factors control showed that 68.5% of these patients met HbA1c <7%, 83.7% of them achieved BP <140/90 mmHg, and 48.2% reached LDL-c <2.6 mmol/L, with only 29.5% of them achieving the three combined therapeutic targets after one-year treatment. We noted that patients with higher levels of baseline HbA1c, older adults, obesity participants, active smokers, patients without exercise, patients with comorbidities, patients with more than one OHA, patients receiving insulin treatment, patients from lower tier hospitals, were less likely to achieve the glycemic control, blood pressure control, lipid control in Chinese patients with newly diagnosed type 2 diabetes based on this study.

This longitudinal cohort study provides valuable information on the current treatment in newly diagnosed type 2 diabetic patients in China, outlining the burdens of glycemic control, blood pressure control and lipid control, identifying gaps in the quality of care and risk-factor control, and revealing the factors influencing these gaps. Continued nationwide evaluation of diabetes control will be important to sustain improvements in care and to minimize the gaps between real-world treatment patterns and clinical guidelines.

## Supplementary information

supplement

## Data Availability

The data that support the findings of this study are available from VitalStrategic Research Institute (Shanghai, China) but restrictions apply to the availability of these data, which were used under license for the current study, and so are not publicly available. Data are however available from the authors upon reasonable request and with permission of VitalStrategic Research Institute (Shanghai, China).
