# Supplementary material for: The risk factors of glycemic control, blood pressure control, lipid control in Chinese patients with newly diagnosed type 2 diabetes _ A nationwide prospective cohort study
Source: Sci Rep. 2019 May 22;9:7709. doi: 10.1038/s41598-019-44169-4 (PMC6531462; doi:10.1038/s41598-019-44169-4)
Supplement: Supplementary file 1 — supplement [file 41598_2019_44169_MOESM1_ESM.docx]

**Title page**

**The risk factors of glycemic control, blood pressure control, lipid control in Chinese patients with newly diagnosed type 2 diabetes _ A nationwide prospective cohort study**

Xiaoling Cai^1^, M.D., Dayi Hu^2^, M.D., Changyu Pan^3^, M.D., Guangwei Li^4^, M.D., Juming Lu^3^, M.D., Qiuhe Ji^5^, M.D., Benli Su^6^, M.D., Haoming Tian^7^, M.D., Shen Qu^8^, M.D., Jianping Weng^9^, M.D., Danyi Zhang^10^, M.D., Jie Xu^10^, Mphill, Linong Ji^1^, M.D.

1. Department of Endocrinology and Metabolism, Peking University People’s Hospital, Beijing, China
2. Department of Cardiology, Peking University People’s Hospital, Beijing, China
3. Department of Endocrinology and Metabolism, Chinese People's Liberation Army General Hospital, Beijing, China
4. Department of Endocrinology and Metabolism, Fuwai Hospital, Beijing, China
5. Department of Endocrinology and Metabolism, The Forth Military Medical University Xi Jing Hospital, Xi An, China
6. Department of Endocrinology and Metabolism, The Second Affiliated Hospital Dalian Medical University, Dalian, China
7. Department of Endocrinology and Metabolism, Sichuan University West China Hospital, Chengdu, China
8. Department of Endocrinology and Metabolism, Shanghai Tenth People's Hospital, Shanghai, China
9. Department of Endocrinology and Metabolism, The Third Affiliated Hospital, Sun Yat-Sen University, Guangzhou, China
10. VitalStrategic Research Institute, Shanghai, China.

Correspondence to:

Linong Ji,

Address: Xizhimen South Street No.11, Department of Endocrine & Metabolism, Peking University People’s Hospital, Beijing, China, 100044;

Tel: +861088324108;

Fax: +861088325534

E-mail address: jiln@bjmu.edu.cn;

Supplementary

# Supplemental Table S1: List of hospitals and doctors participated in this study

**Supplemental eProtocol**

**Supplemental eChecklist**

# Supplemental Table S1: Hospitals included in this study for Patient Recruitment

| **Region** | **Sites** | **Site Name** | **Investigators Name** |
| --- | --- | --- | --- |
| **All Sites** |  | Total Patients |  |
| Northeast | 1801 | Weigong Community Health Service Center of Shenyang Tiexi District | Xianying Liu, Jianbo Zhao |
|  | 2801 | Jilin Electric Power Hospital | Fuxian Liu, Chunna Zhao |
|  | 2802 | People's Hospital of Changchun | Ying Niu, Kaiping Mu, Qi An |
|  | 2803 | Liaoning Diabetes Treatment Center | Bin Gao, Liuli He |
|  | 2804 | The Fifth People's Hospital of Dalian | Rui Yu, Yijun Wang |
|  | 2805 | The First People's Hospital of Dalian Jinzhou District | Shuzhen Wang, Jun Yu |
|  | 3801 | The Second Affiliated Hospital of Dalian Medical University | Benli Su, Lin Zhao |
|  | 3802 | The Third People's Hospital of Dalian | Xiao Zhang, Yanjuan Tian |
|  | 3803 | Jilin People's Hospital | Ying Zhang, Kexin Li |
| North | 1101 | Beijing Xicheng Shichahai Community Health Service Center | Junqing Liu, Lina Zhang |
|  | 1102 | BUPT Community Health Service Center | Xiaoyan Wang, Yanmei Chen |
|  | 2101 | Beijing Haidian Hospital | Wei Huang, Fang Chen, Hongxin Jin |
|  | 2102 | Beijing Hepingli Hospital | Yaping Liu, Heng Quan |
|  | 2103 | Huguosi Hospital of Traditional Chinese Medicine of Beijing University of Chinese Medicine | Lichun Yao, Junqing Liu |
|  | 3101 | Peking University People’s Hospital | Linong Ji, Yingli Chen, Xiaoling Cai |
|  | 3102 | Chinese People's Liberation Army General Hospital（301 Hospital） | Juming Lu, Xiaoman Zou, Fang Chen |
|  | 3105 | Coal General Hospital | Hongmei Li, Yan Zhang |
|  | 3106 | Beijing Fuwai Hospital | Guangwei Li, Yanyan Chen, Xiaoxia Shen |
| East | 1401 | Shanghai Yangpu WujiaochangCommunity Health Service Center | Wei Yuan, Dianhong Li |
|  | 1402 | Shanghai Yangpu yinhangCommunity Health Service Center | Yumei Ma, Xiaoying Dong |
|  | 1403 | Shanghai Yangpu bridge Community Health Service Center | Yuanhong Chen, Hui Liu, Xianping Lin |
|  | 1404 | Shanghai Qingpu Zhaogang Community Health Service Center | Qing Gu, Meijuan Zhu |
|  | 1407 | Nanjing Yijiangmen Community Health Service Center | Fanhong Meng, Bin Liu, Zhuqing Lv, Fang Zhu |
|  | 1408 | Nanjing Ninghai Road Community Health Service Center | Xiaoning Jia, Biwen, Wang |
|  | 2401 | Shanghai Pudong New Area Gongli Hospital | Mingjun Gu, Lianyong, Liu |
|  | 2402 | Nanjing Xuanwu Hospital | Yiqing Yang, Ran Tao |
|  | 3402 | The NO.10 People's Hospital of Shanghai | Shen Qu, Qiong Liao |
|  | 3405 | Jiangsu Provincial Institution Hospital | Rongwen Bian, Yan Hu |
|  | 3407 | Jiulong Hospital of Suzhou | Yanbei Deng, Zongmei Pan |
|  | 3408 | The First People's Hospital of Kunshan | Shao Zhong, Huajin Mao |
|  | 3409 | Suzhou Hospital of Traditonal Chinese Medicine | Fei Huang, Pengcheng Yu |
| Northwest | 1901 | Xi'an Xincheng No.2 Hospital | Yipeng Hou, Bing Xia, Weining Zhang |
|  | 1902 | Xi'an Xincheng Hujiamiao Hospital | Ji Li, Zhigang Zhao, Yonghong Li |
|  | 1903 | Xi'an Xincheng District Hansenzhai Community Health Service Center | Jiang Wu, Ye Liang |
|  | 2901 | Xi'an Xihang Jituan Hospital | Yunan Jia, Wanxia Xiao, Lanying Zhang |
|  | 2902 | Xi'an Huashan Central Hospital | Qingli Fan, Ye Liang |
|  | 2903 | Xi'an Jingyang Yong'an Hospital | Yongyang Wang, Yuanyuan Ren |
|  | 3901 | The Forth Military Medical University Xijing Hospital | Qiuhe Ji, Yujun Li |
|  | 3902 | Chinese People's Liberation Army 451 Hospital | Dafang Wu, Yahong Wang |
|  | 3903 | The Second Hospital of Lanzhou University | Jiangong Ren, Jie Sun |
|  | 3904 | People's Hospital of Gansu | Jing Liu, Suhong Wei |
|  | 3905 | People's Hospital of Xinjiang | Xinlin Wang, Nan Sun |
|  | 3906 | Urumqi Hospital of T.C.M | Yan Xiao, Shikun Luo, Yuhui Wei |
|  | 3907 | Affiliated Hospital of T.C.M of Xinjiang Medical University | Kaili Li, Jianhong Zhu |
|  |  |  |  |
| Southwest | 1601 | Hospital of Southwest Computer Co.,Ltd. | Yana Xiao, Bo Zhang |
|  | 1602 | Xindu Chengxi Community Health Service Center | Xiaohua Wu, Liuli He |
|  | 2601 | The Second Hospital of Chongwing Jiulongpo District | Qiu Xu, Qian Fan |
|  | 2602 | Chongqing Jiangjin Central Hospital | Bin Li, Hongyun Miu |
|  | 2604 | The Seventh Hospital of Chengdu | Hui He, Jingyu Liu |
|  | 2605 | The First People's Hospital of Chengdu Longquanyi District | Li Zhong, Huajin Mao, Jianping Zhong |
|  | 2606 | Pixian People's Hospital | Yumei Zhang, Wei Zhong |
|  | 3601 | The Second Hospital of Chongqing Medical University | Gangyi Yang, Rui Liu |
|  | 3603 | Chongqing Fuling Central Hospital | Rong Xiao, Zehua Zhou |
|  | 3604 | Chongqing Zhongshan Hospital | Song Lu, Shuang Peng |
|  | 3605 | The People's Hospital of Chongqing Changshou District | Qv Hu, Luyan Zhang |
|  | 3606 | Wujing Chongqing General Team Hospital | Yong Liao, Danlan Pu, Hui Wang |
|  | 3607 | No. 416 Hospital of China National Nuclear Corporation | Rong Zhou, Ming Wan, Ting Yao, Jiali Wang |
|  | 3608 | The First People's Hospital of Chengdu | Suyi Liu, Jie Gao |
|  | 3610 | West China Hospital of Sichuan University | Haoming Tian, Jianwei Li, Qilin Liu, Yan He |
|  | 3611 | People's Hospital of Sichuan | Pengqiu Li, Limei Liu, Mingjing Bao, Yan Yang |
|  | 3612 | The Yongchuan Hospital of Chongqing Medical University | Zhiyong Li, Yanmei Chen, Jing Dong |
| South | 1301 | Guangzhou Liwan Caihong Street Community Health Service Center | Yi Lin, Jun Cheng |
|  | 1302 | Guangzhou Haizhu Jianghai Street Community Health Service Center | Zhaoqi He, Jinli Zhu |
|  | 1304 | Hunan Huangxing Hospital | Junjiao Li, Donghui Li |
|  | 1305 | Guangzhou Haizhu District Red Cross Hospital | Liping Li, Yuwei Zhong, Jianghong Liu |
|  | 1306 | Zhengzhou Community Health Service Center Nanyangxincun Office | Mingxun Yin, Xiangmei Ma, Shuhua She |
|  | 2301 | Liwan Hospital | Hongbin Luo, Jianmei Mi |
|  | 2302 | Haizhu Shayuan Street Community Health Service Center | Minlin Liu, Yonglian Xie |
|  | 2303 | Hunan Finance & Trade Hospital | Liping Zhu, Qian Xiong, Lifang Zhong, Bin Huang |
|  | 3301 | General Hospital of Guangzhou Military Command | Aiming Deng, Yidan Liu |
|  | 3302 | Xiangya Hospital | Minxiang Lei, Shaobo Chen, Jing Wang, |
|  | 3303 | The Third Affiliated Hospital of Sun Yat-sen University | Jianping Weng, Weifeng Sun |
|  | 3304 | People's Hospital of Henan | Shilin Wen, Hua Man |
|  | 3305 | The First Affiliated Hospital of Jinan University (Guangzhou Huaqiao Hospital) | Lie Feng, Wanping Xu |
|  | 3306 | The First People's Hospital of Changsha | Jun Ouyang, Jinjin Zhao, Lin Li |
|  | 3307 | Hunan Xiangxi People's Hospital | Lili Chen, Weiwei Yue |
|  | 3308 | Wujing Henan General Team Hospital | Hongbin Liu, Na Lin |
|  | 3309 | The Second Hospital of Zhengzhou University | Suhe Zhang, Yanqin Fu |
|  | 3310 | The First Hospital of Zhengzhou University | Lili Zheng, Zhifang Wang |

**Supplemental eProtocol**

**PROTOCOL**

**Evaluation of Effectiveness of Treatment Paradigm for Newly Diagnosed**

**Type 2 Diabetes Patients in China**

**(NEW2D STUDY)**

***A Study Of China Cardiometabolic Registries (CCMR)***


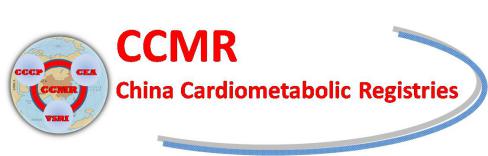


Study Protocol (Version 2.8)

May 21, 2012

Proposed by:

**Linong Ji, MD on behalf of CCMR Advisory Board**

Co-Chairman of CCMR Advisory Board

Director of Endocrinology & Metabolism

People’s Hospital of Peking University

**Danyi Zhang, MD on behalf of CCMR Executive Committee**

Chairman of CCMR Executive Committee

Chief Scientific Officer

VitalStrategic Research Institute

SYNOPSIS

| Study Type | Observational, longitudinal, prospective cohort study |
| --- | --- |
| Study Subjects | Newly diagnosed type 2 diabetic (T2D) patients defined as diagnosis of T2D according to the Chinese Guideline (2010) for Type 2 Diabetes, within the past 6 months. |
| Number of Subjects | Approximately 5000 patients from nation-wide hospitals will be recruited |
| Study Location | Approximately 80 hospitals (40 tier 3, 25 tier 2, 15 tier 1) from 6 major geographic regions in China will be recruited for this study. |
| Principal Investigator | Prof Linong Ji, Director of Endocrinology Department, Beijing University, People’s Hospital, Beijing, China |
| Investigators | Qualified internists and endocrinologists will be selected from the members of CEA and other CCMR physician networks. |
| Objectives | Primary objectives:  To assess the evolution of treatment patterns for newly diagnosed T2D patients and the clinical outcomes during 12-month follow up.  Secondary objectives:   1. To evaluate the reasons and the process of each step of the changes in treatment regimen during the 12-month follow up. 2. To evaluate the influential factors related to the changes of the treatment patterns for newly diagnosed T2D, including clinical outcomes, pattern of blood glucose monitoring, social-economical status (*i.e.* insurance status, income status, education background, *etc*), patients’ compliances, patients’ knowledge of diabetes, hospital tier, specialities of the physicians; 3. To assess the incidence of CVD risk factors (*i.e.* hypertension, lipidemia and obesity) and the intervention patterns; 4. To assess the associated incidence of diabetic complications, including cardiovascular / cerebrovascular and microvascular diseases; 5. To assess the application of diabetes related clinical examinations such as Fundus examination and diabetic foot screening; 6. To assess the methods of diagnosis (*i.e.* routine physical examination, health screening, seeking health care due toT2D symptoms, seeing doctors for other diseases, *etc*), and location of diagnosis (*i.e.* the tier of hospital, the physician specialty, *etc*) and the influential factors. 7. To assess the incidence of hypoglycemia and other AEs and SAE, that may be related to anti-diabetes medications. 8. To access the medical resource consumption pattern for treating diabetes. 9. To evaluate the health conditions and quality of life at the baseline and at the 12^th^ month. |
| Study Design | Newly diagnosed (within 6 months) type 2 diabetic patient who is visiting tier 3, 2, or one hospital, may be considered for enrollment in this study, as long as the patient meets the inclusion and exclusion criteria and is willing to sign on the informed consent form.  Enrolled patients will receive routine treatment (diet & exercise or medication) and will be asked to return to see the same physicians for follow up visits at 3, 6. 9 and 12 month after the first visit.  Following measurements will be recorded, laboratory reports will be obtained.  Baseline measurement only:   - Demography - Medical history and co-morbidity - Diabetes history and related symptoms - Diabetic complications - Family history - Education level and knowledge about T2D - Social-economic background - Diagnosis methods and information of the health care providers - Questionnaire for diabetes knowledge assessment - Quality of life assessment (repeated at the 12 month visit)   Baseline and follow up visits   - Blood pressure - Health behavior (Smoking history, exercise pattern) - Physical exam (height, body weight, waist circumference) - Medication (including herbal medicine, generic name, brand name, dosage, frequency, years taken, purpose, reason for switch of medication, if necessary) - Hypoglycemic symptoms and severity - Lab tests: - Serum glucose - HbA1c - Lipid panel (Total cholesterol, triglyceride, LDL, HDL) - Kidney function (serum creatinine) - Liver function (Gamma-GT, ALT, AST) - Urine analysis (albumin, creatinine or albumin to creatintine ratio)   If any additional diagnostic test is ordered as part of the routine medical course, a copy of the results should be obtained. Any change of medical history, hospitalization, and medication will be specially noted (cause, dosage, outcome, etc).  Notice:   1. Lipid panel and liver function are tested every 6 months, other lab tests are conducted every 3 months 2. If the patients have extra visits or examinations between any two scheduled visits, the reasons for these visits and the related treatment and test results should be recorded 3. If there are changes of disease conditions, hospitalizations or medications between any two scheduled visits, the related details should be recorded (reasons, dosages, results, *etc*) |
| Inclusion and Exclusion Criteria | Inclusion criteria   - 20 years of age or older - Confirmed diagnosis of type 2 diabetes made within past 6 months - Willing to return for all follow up visits (3, 6, 9, 12 months after baseline visit)   Exclusion criteria  To help ensure sufficient rate of return for follow up in the longitudinal study, the exclusion criteria are the following:   - Patients who are currently pregnant or are breast feeding, or plan to get pregnant during the following year; - Patients who are not willing or not able to return to the same hospital for follow up visits every 3 months for one year after enrollment; - Patients who are participating in any other interventional clinical trials; - Patients who have been on diabetes medications but have no clear information regarding the medication used; - Patients who are not willing to sign informed consent form. |
| Observational endpoints | **Primary endpoint**  The overall proportion of patients whose HbA1c is less than 7%, measured at the end of one year follow up.  **Secondary endpoints**   1. The proportions of patients receiving one OAD, two OADs, > two OADs, insulin alone, insulin+one OAD, insulin+two OADs, insulin+>two OADs and by class of OADs at 3, 6, 9, and 12 months; 2. Mean duration of diet & exercise alone (t4), initial anti-diabetic treatment (ADT) (t1), initial ADT is switched or 2nd ADT is added (t2), 2nd ADT is switched or 3rd ADT is added (t3) ; (Figure 1 Treatment Pattern Diagram); 3. The proportion of patients reached the therapeutic target of glycemic control by treatment regimen and by OAD class at 3, 6, 9 and 12 months: 4. The correlation between treatment patterns and patient demographics, social-economic status, insurance, medical history, HbA1C level, type of hospital, physician years of practice, etc; 5. Incidence of microvasular diabetic complication: diabetic retinopathy, nephropathy, neuropathy, and macrovascular complications: cardiovascular events, peripheral vascular complications, and stroke, at 3, 6, 9, and 12 months; 6. Were the diabetes related clinical examinations applied such as Fundus examination and diabetic foot screening; 7. Incidence of hypoglycemia associated with various ADTs; 8. Diagnosis patterns, including the methods of diagnosis (i.e. routine physical examination, seeing doctors for other diseases, etc), and type of hospital, as well as its association with demographics and regions of residence. |
| Data Collection and Quality Control | The study will be conducted in accordance with ICH guidelines and GCP standard. Investigators will report all data specified by the protocol by using a web-based electronic CRFs.  A web-based study center will be used to provide investigators 24/7 support on conducting the study, which will provide frequent patient enrollment updates, study materials, and other informative material (*e.g.* publication of key International literature, trend and dynamics in the outcomes research area *etc.*)  Data quality control will include the following steps:   - Automatic edit check at data entry on the EDC system - Real time data query created by the EDC-attached data management system; - 100% site monitoring ensuring adequate patient enrollment, completion of original medical chart and documentation, and protocol compliance; - 50% remote source document verification (rSDV) and 10% on-site SDV at each site; - Frequent data query from a specific site will trigger a data audit visit at the site. Random site audits will also be performed to ensure proper study conduct; - Lab evaluations will be completed at all sites before the enrolment starts. |
| Sample Size and Statistic Evaluation | As this is a descriptive study, there will be no hypothesis testing. The sample size will be calculated based upon a desired margin of error for the proportion of subjects reaching the target HbA1c level.  According to the results of the Polish ARETAEUS1 study, 28.9% of the patients who were diagnosed with T2D within 2 years achieved a therapeutic target of HbA1c <6.5%. Pan et al reported 25.9% of T2D patients in China achieved a target glycemic control level of HbA1c <6.5%. A recently completed CCMR study showed that 30.7% of the stabilized T2D patients (diagnosed more than 6 months) in China reached this therapeutic target. We thus estimate that the proportion of newly T2D patients reaching the target of HbA1c < 6.5% in this study is approximately between 26%-31%.  Assuming a confidence level of 95%, a confidence interval of 0.05 (margin of error of +/- 2.5%), and the expected proportion (p) of 0.30, n=1,315 subjects are required. To ensure each of the three hospital tier has sufficient subjects to demonstrate the assumed proportion of patients reaching the target HbA1c <6.5%, the total sample size would be n=3,945 (1,315 x 3). Attrition is often a challenge in observational studies and three recent, large population-based observational studies of diabetes patients in China reported attrition rates ranging from 14% to 25%. Assuming an attrition rate of approximately 20% for this study, a sample size of 4,931 is required.  Demographics and baseline characteristics will be summarized. Multivariate regression analysis will be performed to identify independent predictor of treatment outcomes. Specific subgroups will be compared with respect to their baseline characteristics, diabetes history, cardiovascular risk factors for glycemic control, clinical outcomes, and vascular events. Cox hazard regression model or logistic regression model will be used to assess relative risks of baseline characteristics or other factors, by using incidence of inadequate glycemic control as dependent variables and baseline risk factors as independent variables.  An interim analysis of the baseline data will be performed upon completion of patient enrollment, in addition to the final study analysis.  For diabetes knowledge assessment and EQ-5D questionnaire, the results will be analyzed using descriptive statistics; comparative analysis will be conducted between the baseline EQ-5D and the last visit results. |
| Study Duration | Study preparation (EC/IRB approval): 3 months  Patient enrollment period: FPI to LPI = 6 months  Follow up period: 12 months  Data Cleaning, analysis and reporting: 3 months  Total duration of the study: 24 months (from commencement to CSR) |
| Study Organization | Study Funder: Bristol Myers Squibb  Study Sponsor: VitalStrategic Research Institute  Advisory Board: CCMR Advisory Board  Study Steering Committee: National and regional level medical experts recommended by the CCMR Advisory Board. |
| Study Management | Study management will be provided by CCMR Study Center , VitalStrategic Research Institution, who will be responsible for   - Study protocol, CRF, ICF development - Coordinate with steering committee and investigators - Qualifying investigators and gain EC approval - Investigator meeting and training (on protocol and on outcomes research concept) - Study initiation, monitoring, auditing and closing - EDC design and maintenance - Handling safety reports related to BMS marketed products - Data management and statistic analysis - Study reporting - Design and maintaining Web-Based Study Center - Project management - Abstract and manuscript preparation |
| Study Contact | Danyi Zhang, MD, MS Chief Medical Officer VitalStrategic Research Institute [dzhang@vitalstrategic.com](mailto:dzhang@vitalstrategic.com) 1055 Westlakes Drive, Suite 300 Berwyn, PA, 19312 USA Tel: +1 610 727 4550 Fax: +1 610 727 4001 Mobile: 610 864 1415  Shanghai VitalStrategic Research Institute 445 Jiang Ning Road, ShiMei Building, Suite 16D Shanghai, 200041 China Tel: +86 21 6271 5538 Fax: +86 21 6271 5668 |

Table of Contents

[Proposed by: 1](#_Toc324850819)

[SYNOPSIS 2](#_Toc324850820)

[1. BACKGROUND 11](#_Toc324850821)

[2. INTRODUCTION 13](#_Toc324850822)

[3. STUDY OBJECTIVES 14](#_Toc324850823)

[3.1. Primary Objectives: 14](#_Toc324850824)

[3.2. Secondary Objectives: 14](#_Toc324850825)

[4. STUDY DESIGN 15](#_Toc324850826)

[4.1. General design 15](#_Toc324850827)

[4.2. Inclusion criteria 15](#_Toc324850828)

[4.3. Exclusion criteria 15](#_Toc324850829)

[5. STUDY PROCEDURE 16](#_Toc324850830)

[5.1. Baseline Measurements 16](#_Toc324850831)

[5.2. Measurements at Baseline and Follow-up Visits 17](#_Toc324850832)

[5.3. Study Flow Chart 21](#_Toc324850833)

[6. OBSERVING ENDPOINTS 22](#_Toc324850834)

[6.1. Primary endpoints 22](#_Toc324850835)

[6.2. Secondary endpoints 22](#_Toc324850836)

[7. SAMPLE SIZE AND STATISTIC EVALUATION 24](#_Toc324850837)

[8. INFORMED CONSENT 25](#_Toc324850838)

[9. MANAGEMENT REQUIREMENTS 25](#_Toc324850839)

[9.1. Data collection 25](#_Toc324850840)

[9.2. Quality Control 26](#_Toc324850841)

[9.3. Study Duration 26](#_Toc324850842)

[9.4. Study Structure 27](#_Toc324850843)

[10. ADVERSE EVENT REPORTING 27](#_Toc324850844)

[10.1. Investigators’ responsibilities 27](#_Toc324850845)

[10.2. Definitions of adverse events 27](#_Toc324850846)

[11. PUBLICATION OF THE RESULTS 29](#_Toc324850847)

[12. COMPLIANCE 29](#_Toc324850848)

[13. ETHICAL REVIEW 29](#_Toc324850849)

[14. REFERENCE: 30](#_Toc324850850)

# BACKGROUND

The prevalence of diabetes around the world has increased from 30 million in 1985 to the current 235 million, and is expected to grow to 300 million in 2030, with more than 75 percent occurring in countries such as India and China [1]. In China, economic prosperity and changes in diet and lifestyles have resulted in steep increase in prevalence of overweight, obesity, and diabetes. According to the latest study, in adults of 20 years of age or older, the incidence of diabetes has reached 9.7% and the prevalence 92.4 million, making China the country with the highest prevalence in the world [1]. Reports also indicate that more than 60% of the diabetes in China are not diagnosed and that the prevalence of pre-diabetes is just as high as that of diabetics, exposing China to a sever social economic burden [2-4]. According to the WHO [5], the cost for managing diabetes around the world has already risen to represent 11.2% of the total healthcare cost, and in China, between 2005-2015, the cost for managing diabetes may be as high as 55 billion US dollars. Type 2 diabetes mellitus is associated with an increased risk for both micro-and macrovascular complications, and cardiovascular diseases (CVD) are the most common causes of death among these patients [6-7]. In patients with coronary artery disease (CAD), patients with diabetes are associated with more severe and diffuse atherosclearotic diseases and higher mortality compared to non-diabetic patients [8]. In a nationwide, cross over cohort study that was recently completed in China [9], the results showed that the duration of diabetes is positively correlated with the incidence of CAD and all microvascular diabetic complications. Preventing diabetes progression and preventing the onset of cardiovascular diseases early on at the stage of newly diagnosis is thus one of the most important goals in managing type II diabetes [10-11].

Long-term follow results from DCCT and UKPDS (United Kingdom Prospective Diabetes Study) showed that intensive glucose control strategy at the early stage of diabetes treatment could significantly reduce the risks of diabetic complications of small and large blood vessels, indicating that standard diagnosis and treatment implementation together with reaching the treatment target are the key measures to reduce the risks of diabetic complications and improve the prognosis for newly diagnosed diabetes patients.

According to the Chinese Guidelines for Prevention and Treatment of Diabetes [12], the therapeutic target for type 2 diabetes is defined as HbA1C < 7.0%, which is consistent with ADA recommendations[13]. However, based on various guidelines and UKPDS study results as well as long-term follow-up visits of the UKPDS patients, it was believed that the glycemic control for newly diagnosed type 2 diabetes patients without severe complications should be stricter, such as HbA1c<6.5% or close to normal. Various national surveys conducted in China in 2003, 2004, 2006 have shown that only about one forth of type 2 diabetes patients in China reached this therapeutic target [14-17]. Likewise, the implementation of standard diagnosis and treatment procedure for diabetic complications as well as the control of cardiovascular risk factors are still unsatisfactory. In general, the current management of diabetes in China has a huge gap between the guideline requirements and the actual outcomes. Currently, the exact reasons leading to the gap are not clear, probably it’s associated with the knowledge and skills of physicians, the patients’ compliance, medical insurance, drug selection and limitations, and the monitoring of the essential indications. To improve the level of management for type 2 diabetes in China, we urgently need to explore the specific reasons for this gap, develop specific plans that set to reduce the gap, and to carry out translational medical research to find out actionable solutions.

Over the recent few years, a number of new antidiabetic treatments, such as DPP-IV inhibitors, have been introduced to the real world practice of diabetes management in China, creating exciting opportunities to potentially improve glycemic control. UKPDS provided the evidence for improving glycemic control [18] through aggressive treatment in western population. On the other hand, it is believed that not all patients have benefited from current pattern of diabetes management in China, partially due to diversity of standards of clinical practices and complexity of diabetes care itself. For example, it is unclear which treatment regimen works more effectively for managing newly diagnosed type 2 diabetes patients and whether a more aggressive strategy should be used during the early stage of treatment. Moreover, it remains undocumented that what factors other than medications may influence the treatment outcomes. There are clearly significant unmet needs in managing diabetes in China and thus significant opportunities to leverage the existing medical technology.

This study is thus designed to evaluate current treatment patterns and treatment outcomes for managing newly diagnosed type 2 diabetes patients and to assess specific unmet needs, influencing factors, management platforms, and gaps and trends in practice, providing a comprehensive knowledge in current management of newly diagnosed diabetes and insight on opportunities and strategy for future research and development of improved therapy.

# INTRODUCTION

The NEW2D study is proposed to be conducted in collaboration with the Advisory Board of China Cardiometabolic Registries (CCMR) and VitalStrategic Research Institute.

CCMR is one of the largest registry/cohort study programs ever being organized in China. It is designed to better understand the epidemiology and unmet medical needs in major cardiovascular and metabolic diseases, better establish evidence of clinical outcomes, better define gaps in medical guidelines, better enhance the values of innovative medical therapy and diagnostics, and ultimately better improve the quality of patient care. CCMR is organized by Chinese Medical Doctor Association (CMDA) - Chinese College of Cardiology Physicians (CCCP), CMDA-Chinese Endocrinologists Associations (CEA), and VitalStrategic Research Institute (VSRI). It is governed by an advisory board that is comprised of internationally renowned opinion leaders from Europe, US, and China. The program was launched in October 2008. When fully executed, it will include a series of individually designed cohort studies in all major cardiovascular diseases such as acute coronary syndromes, hyperlipidemia, hypertension, atrial fibrillation, heart failure, diabetes. CCMR currently have 3 cohort studies on going, and several posters have been presented at American Diabetes Association, American College of Cardiology and other international conferences. All CCMR studies are registered at [www.clinicaltrials.gov](http://www.clinicaltrials.gov).

While CCMR is expected to generate information about prevalence, clinical outcomes, healthcare patterns, and therapeutic gaps in China that was never previously well documented, it is also expected to generate valuable information on a global scale in respect to disease progression, disease management, and unmet needs. Taking the advantage of large patient source, relatively lower operating cost, and strong commitment of clinicians in population based research, CCMR is expected to make significant and unprecedented contributions to the worldwide cardiovascular and metabolic research.

BMS has been a supporter of the CCMR program since its inception and has provided CCMR a meeting sponsorship for one of its symposia on Cardiovascular and Metabolic Diseases held in 2009 in China. CCMR has recognized BMS’ commitment in improving diabetes management in China and valued the collaboration in the design of this study.

VitalStrategic Research Institute (VSRI) is a research organization focusing on evidence-based clinical, epidemiological, and outcomes research. It collaborates with worldwide prestigious institutions and organizations such as Harvard Medical School, Fudan University, and China Medical Doctor Association, to initiate epidemiology and outcomes research aiming to enhance evidence based medicine and improve health care outcomes; it partners with biopharmaceutical and medical device companies such as Merck, Johonson & Johonson, and Roche, to conduct Phase II-IV clinical research aiming to better define unmet medical needs and enhance the values of medical solutions and products. . VSRI is a co-founder of CCMR and has been designated by CCCP and CEA to be responsible for the execution of CCMR studies. VSRI is based in Philadelphia with operating office in Shanghai, China. More information about VSRI can be found at [www.vitalstrategic.com](http://www.vitalstrategic.com).

# STUDY OBJECTIVES

## Primary Objectives:

To assess the evolution of treatment patterns for newly diagnosed T2D patients and the clinical outcomes during a 12-month follow up.

## Secondary Objectives:

1. To evaluate the reasons and the process of each step of the changes in treatment regimen during the 12-month follow up.
2. To evaluate the influential factors related to the changes of the treatment patterns for newly diagnosed T2D, including clinical outcomes, pattern of blood glucose monitoring, social-economical status (i.e. insurance status, income status, education background, etc), patients’ compliances, patients’ knowledge of diabetes, hospital tier, specialties of the physicians;
3. To assess the incidence of CVD risk factors (i.e. hypertension, lipidemia and obesity) and the intervention patterns;
4. To assess the associated incidence of diabetic complications, including cardiovascular / cerebrovascular and microvascular diseases;
5. To assess the application of diabetes related clinical examinations such as Fundus examination and diabetic foot screening;
6. To assess the methods of diagnosis (i.e. routine physical examination, health screening, seeking health care due toT2D symptoms, seeing doctors for other diseases, etc), and location of diagnosis (i.e. the tier of hospital, the physician specialty, etc) and the influential factors.
7. To assess the incidence of hypoglycemia and other AEs and SAE, that may be related to anti-diabetes medications.
8. To access the medical resource consumption pattern for treating diabetes.
9. To evaluate the health conditions and quality of life at the baseline and at the 12th month.

# STUDY DESIGN

## General design

Any newly diagnosed (within 6 months) type 2 diabetic patient who is visiting tier 3, 2, or 1 hospitals, may be considered for enrollment in this study, as long as the patient meets the inclusion and exclusion criteria and is willing to sign on the informed consent form.

Enrolled patients will receive routine treatment (diet & exercise or medication) and will be asked to return to the same investigator for follow up visits at 3, 6, 9 and 12 month after the baseline visit.

## Inclusion criteria

- 20 years of age or older;
- Confirmed diagnosis of type 2 diabetes made within past 6 months according to the 2010 Chinese Guideline for Type 2 diabetes;
- Willing to return for all follow up visits (3, 6, 9 and 12 months after baseline visit).

## Exclusion criteria

- Patients who are currently pregnant or are breast feeding, or plan to get pregnant during the following year;
- Patients who are not willing or not able to return to the same hospital for follow up visits every 3 months for one year after enrollment (e.g. do no reside locally);
- Patients who are participating in another interventional clinical trials;
- Patients who have been on diabetes medications but have no clear information for the drugs used;
- Patients who are not willing to sign informed consent form.

# STUDY PROCEDURE

## Baseline Measurements

- Demography: Age, gender, residential region.
- Diabetes history and related symptoms: Duration or diagnosis (must be < 6 months), how was diagnosis made (using the 2010 Chinese Guideline for Diabetes Prevention and Treatment [19]) , signs and symptoms.
- Diagnostic pathways:
  1. Seeking health care due to diabetic symptoms
  2. Already found an increased blood glucose once, and confirmed by a second test
  3. Seeking health care due to other diseases
  4. Self-demanded lab tests
  5. Participating in an epidemiological study / campaign
- Screening methods for diabetes (could be singular or multiple choices):
  1. OGTT test
  2. “Bun test”
  3. Urine glucose
  4. Others
- Diagnostic methods for diabetes* (reference table 1), diabetic symptoms plus:
  1. Random blood glucose (the blood glucose level at any time during a day without knowing the time of last meal)
  2. FBG (fasted for at least 8 hours without calories intake)
  3. 2-hour postprandial blood glucose
- Diabetics symptoms: thirsty, constant hunger, frequent micturition, obvious weight loss, fatigue, prone to inflammation, blurred vision, numbness/pain, leg or foot ulcers, skin itching, acute complications such as ketosis, hyperosmolar coma, *etc.*
- The hospitals and departments where diabetes was diagnosed:
  1. Tier 1 hospital
  2. Tier 2 hospital: endocrinology dept., internal medicine dept.
  3. Tier 3 hospital: endocrinology dept., internal medicine dept.
- Disease history:
  1. Diabetic complication history: the acute or chronicle diabetic complications at or after the diagnosis, including ketosis, hyperosmolar coma, HHS, micro blood vessels lesions (retinal lesions, nephrological diseases, peripheral neuropathy).
  2. Medical history and co-morbidity: Including any major medical procedure or surgery occurred within the last 12 months, any major co-morbidity (e.g. cardiovascular disease, renal disease, other diabetic complications, cancer, etc).
- Family history: Family history of diabetes and cardiovascular diseases
- Education level and knowledge about T2D: Highest formal education received as well as response to simple questionnaire assessing the level of knowledge about T2D.
- Social-economic background: Employment status, marital status, monthly income, insurance type and coverage.
- Questionnaire for diabetes knowledge assessment
- Quality of life assessment (repeated at the last visit)

## Measurements at Baseline and Follow-up Visits

Each patient will return to see the same physicians for follow up visits at 3, 6. 9 and 12 month after the first visit, and the following measurements will be recorded, laboratory reports will be obtained.

- Blood pressure: The subjects should be resting for at least 5 minutes prior to measurement of blood pressure. Measure the sitting or supine blood pressure, and take the mean of values of 2 continuous measurements. There should be 2 minutes interval between the 2 measurements.
- Height and body weight: Remove the shoes and socks prior to measurement, as well as wear light clothes. Formula for body mass index calculation: BMI= Body weight (kg)/Body height^2^ (m)
- Waist circumference: Remove the clothes on the waist and stand relaxed with both hands down naturally. Then use tape around the waist and adjust its location to make the tape pass the intermediate points between superior margins of hip bones at both sides and lower costal margins. Pay attention to that the tape should be parallel to the floor and cling to but no compress the skin.
- Health behavior
- Smoking history: Record the average number of cigarettes per day and smoking time. A smoker is defined as a person who smokes 1 cigarette a day on the average for at least 1 year.
- Exercise pattern: Physical inactivity means the subjects do not have frequent physical exercise (such as amount of exercise equal to that in walking for at least 20-30 minutes a day, 3-4 days a week).
- Medication: Including medication previously prescribed for glycemic control and any other concurrent medications. Record generic name, brand name, dosage, frequency, years taken, and purpose. Current and any change of medications since last recorded visit for treatment of type 2 diabetes, diabetic complications, lipid-regulation, and hypertension, including prescription drugs, OTC drugs and Chinese herbal medicines should be recorded.
- Reason for adjusting anti-diabetic medications
  1. Unsatisfactory treatment outcomes: according to FBG results, or 2-hour postprandial blood glucose, or HbA1c
  2. Adverse events: hypoglycemia, gastrointestinal reactions, edema, weight gain, change of liver or kidney functions, allergic, other discomforts.
  3. Economical reasons
  4. Self-adjustment: details will be required
- Compliances: does the patient routinely take medicines in accordance with the prescriptions, if not, the specific reasons will be required
- Hypoglycemic symptoms and severity: Hypoglycemia is defined as the fasting plasma glucose level below 70 mg/dL according to the American Diabetes Association 2009 Position Statement [12]. The symptoms of hypoglycemia include but not limited to sweating, hunger, trembling, anxiety, confusion, blurred Vision. A simple questionnaire will be used to record patient reported symptoms and severity.
- Lab tests: Blood tests should be performed after fasting for at least 10 hours.
- Serum glucose: The oxidase method should be used;
- HbA1C: It should be determined by high performance liquid chromatography (HPLC)
- Lipid panel: TC – total cholesterol, LDL-C – low density lipoprotein, TG – triglyceride, HDL-C – high density lipoprotein
- Kidney function: serum creatinine
- Liver function (Gamma-GT, ALT, AST)
- Urine test (urine albumin, creatinine or albumin / urine creatinine ratio)

Notice: Lipid panel and liver function are tested every 6 months, while other lab tests are conducted every 3 months.

If any additional diagnose test is ordered as part of the routine medical course, a copy of the results should be obtained. Any change of medical history, hospitalization, and medication will be specially noted (cause, dosage, outcome, etc). Unsolicited safety reports on products marketed by Bristol Myers Squibb will be collected.

Lab test methods:

- - - All blood samples should be venous blood after at least10-hour fasting;
    - HbA1c should be tested using high performance liquid chromatography (HPLC) method;
    - Fasting blood lipids: total cholesterol, triglycerides, high density lipoprotein, low density lipoprotein;
    - Urinalysis: 24 hours urine, short time urine, or random urine are accepted;
    - Albumin to creatinine ratio: urine (3-4 ml) measured within two hours after collection. Urine albumin is tested using immune turbidimetric method. Urine creatinine is tested using oxidase method.
- In addition to the scheduled follow-up visits of NEW2D and the information collected at each visit, the extra visits of the patients should also be briefly recorded, including the date, the reason (needed prescription, needed treatment), having tested for blood glucose, method of the test and the results if available;
- Adverse events: gastrointestinal reactions, edema, weight gain, change of liver or kidney functions, allergic, other discomforts (details);
- Severe adverse events: for BMS products only.
- Self-evaluated life quality is collected with EQ-5D questionnaire at baseline and last visit[21].

## Study Flow Chart

| **Procedure** | **Baseline Visit** | **Follow Up Visits (3, 6, 9, and 12 months after baseline visit +/- one month)^g^** |
| --- | --- | --- |
| Inclusion/Exclusion Criteria | X |  |
| Written informed consent form | X |  |
| Demographic Data ^a^ | X |  |
| Diabetes history | X |  |
| Family history | X |  |
| Medical history and co-morbidity ^b^ | X | X |
| Knowledge about T2D | X | X |
| Health behavior ^c^ | X | X |
| Physical Exam^d^ | X | X |
| Medication ^e^ | X | X |
| Hypoglycemic symptoms and severity | X | X |
| Laboratory tests ^f^ | X | X |
| Unsolicited safety report ^h^ | X | X |
| Self-evaluated life quality ^i^ | X | X |

1. Demographic data include age, gender, residential region, educational level, and social-economic status.
2. Including cardiovascular event and other complication occurred as medical history or during follow up.
3. Including smoking history and exercise pattern.
4. Including height, body weight, waist circumference, blood pressure
5. Current and any change of medications since last recorded visit for treatment of type 2 diabetes, diabetic complications, lipid-regulation, and hypertension, including prescription drugs, OTC drugs and Chinese herbal medicines with their names and doses.
6. Laboratory tests include: fasting blood glucose, HbA1c, fasting blood lipid (TC, HDL-C, LDL-C, and TG, tested every 6 months), liver function (every 6 months), kidney function (serum creatinine), and urine analysis (microalbumin/albumin, urine creatinine, or albumin to creatinie ratio).
7. An one month time window is permitted for each follow up visit.
8. Adverse events include gastrointestinal reactions, edema, weight gain, liver and kidney function, allergies, etc.
9. Unsolicited safety reports on products marketed by the BMS will be collected, then reported to health bureau and BMS
10. EQ-5D questionnaire will be applied.

# OBSERVING ENDPOINTS

## Primary endpoints

The overall proportion of patients whose HbA1c is less than 7%, measured at the end of one year follow up.

## Secondary endpoints

The secondary endpoints of this study include the following;

1. The proportions of patients receiving one OAD, two OADs, > two OADs, insulin alone, insulin+one OAD, insulin+two OADs, insulin+>two OADs and by class of OADs at 3, 6, 9, and 12 months;
2. Mean duration of diet & exercise alone (t_4_), initial anti-diabetic treatment (ADT) (t_1_), initial ADT is switched or 2^nd^  ADT is added (t_2_), 2^nd^ ADT is switched or 3^rd^ ADT is added (t_3_) ; (Figure 1 Treatment Pattern Diagram);
3. The proportion of patients reached the therapeutic target of glycemic control by treatment regimen and by OAD class at 3, 6, 9 and 12 months:
4. The correlation between treatment patterns and patient demographics, social-economic status, insurance, medical history, HbA1C level, type of hospital, physician years of practice, etc;
5. Incidence of microvasular diabetic complication: diabetic retinopathy, nephropathy, neuropathy, and macrovascular complications: cardiovascular events, peripheral vascular complications, and stroke, at 3, 6, 9, and 12 months;
6. Were the diabetes related clinical examinations applied such as Fundus examination and diabetic foot screening;
7. Incidence of hypoglycemia associated with various ADTs;
8. Diagnosis pattern: to assess the methods of diagnosis (i.e. routine physical examination, seeing doctors for other diseases, etc), and location of diagnosis, demographic information of the patients and the regions of residence;
9. Quality of life assessment (baseline and the last visit);
10. Resource consumption patterns based on the information collected on the cost of medications, cost of diagnostic test and other relevant [procedure.

Figure 1 Treatment Pattern Diagram


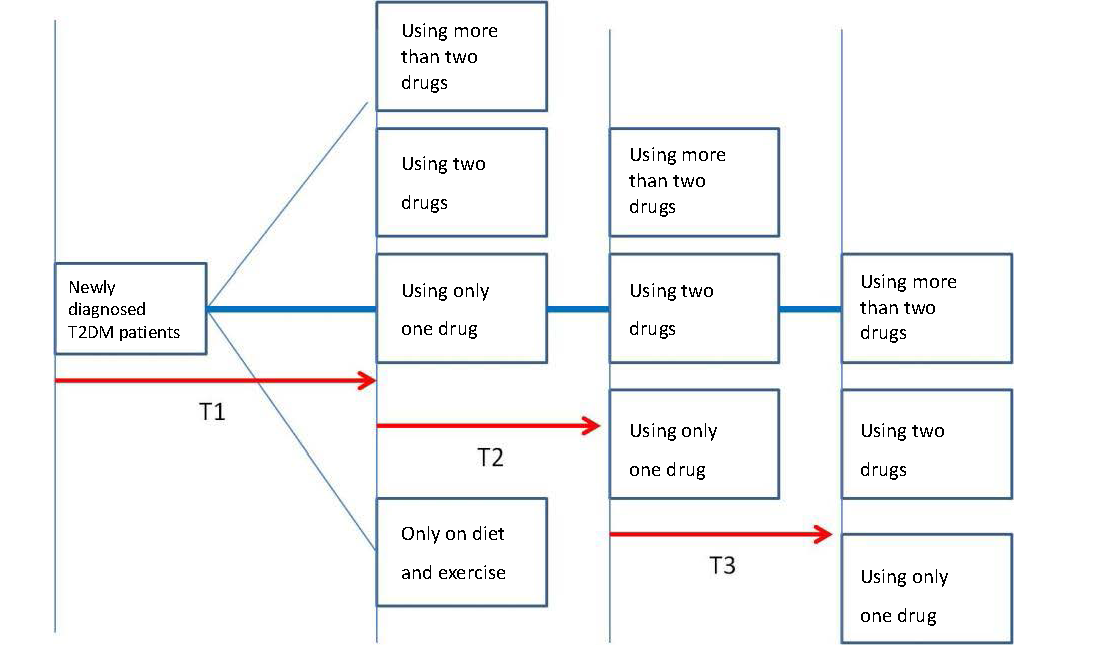


T1 = duration between diagnosis and initiation of (single) treatment regime， T2 = duration between initiation of single treatment regime and duple regime， T3 = duration between initiation of duple treatment regime and multiple regime

# SAMPLE SIZE AND STATISTIC EVALUATION

As this is a descriptive study, there will be no hypothesis testing. The sample size is calculated based upon a desired margin of error for the proportion of subjects reaching the target HbA1c level.

In the 2007 Chinese Guideline for Diabetes Prevention and Treatment [20], the target HbA1c level was set to be <6.5%,. However, in the newest Chinese Guideline published in November 2011 [19], the target HbA1C level has been adjusted to <7.0%, based on the evidence showing that HbA1c<7.0% is already associated with decreased incidence of diabetic complications and that further reduction of HbA1c with using more aggressive intervention might result in an increased incidence of hypoglycemia. In patients with cardiovascular complications, further reduction of HbA1C beyond 7.0% may even be associated with increased risk of CV events. On the other hand, for newly diagnosed patients, the latest guideline still recommended to aim for normal level of HbA1C (<6.5%) by using insulin or other ADT (anti-diabetic treatment) with low risk of hypoglycemia. The target HbA1C level for this study is thus set to be consistent with the guideline, i.e. 6.5%.

According to the results of the Polish ARETAEUS1 study [19], 28.9% of the patients who were diagnosed with T2D within 2 years achieved a therapeutic target of HbA1C <6.5%. Pan et al [14] reported 25.9% of T2D patients in China achieved a target glycemic control level of HbA1C <6.5%. A recently completed CCMR study showed that 30.7% of the stabilized T2D patients (diagnosed more than 6 months) in China reached this therapeutic target. We thus estimate that the proportion of newly T2D patients reaching the target of HbA1c < 6.5% in this study is approximately between 26%-31%.

Assuming a confidence level of 95%, a confidence interval of 0.05 (margin of error of +/- 2.5%), and the expected proportion (p) of 0.30, n=1,315 subjects are required. To ensure each of the three hospital tier has sufficient subjects to demonstrate the assumed proportion of patients reaching the target HbA1c <6.5%, the total sample size would be n=3,945 (1,315 x 3). Attrition is often a challenge in observational studies and three recent, large population-based observational studies of diabetes patients in China reported attrition rates ranging from 14% to 25%. Assuming an attrition rate of approximately 20% for this study, a sample size of 4,931 is required.

Demographics and baseline characteristics will be summarized. Multivariate regression analysis will be performed to identify independent predictor of treatment outcomes. Specific subgroups will be compared with respect to their baseline characteristics, diabetes history, cardiovascular risk factors, clinical outcomes, microvascular and macrovascular events. Cox hazard regression model or logistic regression model will be used to assess relative risks of baseline characteristics, cardiovascular risk factors, and other independent factors by using incidence of inadequate glycemic control as dependent variables. An interim analysis of baseline data will be performed upon completion of patient enrollment, in addition to the final study analysis.

For diabetes knowledge assessment and EQ-5D questionnaire, the results will be analyzed using descriptive statistics; comparative analysis will be conducted between the baseline EQ-5D and the last visit results.

# INFORMED CONSENT

The subjects (or legal representatives of the subject) should sign the informed consent form on the first visit after fully understand of the purpose and procedure of the study. The patients should be aware that they should return for follow up visits every 3 months for one year for physical and laboratory evaluations.

# MANAGEMENT REQUIREMENTS

## Data collection

The study will be conducted in accordance with ICH guidelines and GCP standard. Investigators will first collect all original patient information by using a “Medical Record Worksheet”, which will be served as “source information” and will be kept and maintained by the investigators. As a second step, the investigators will report all data specified by the protocol onto a web-based electronic CRFs by using an EDC system (VitalEDC, VitalStrategic). Data query report will be generated automatically in real time.

A web-based study center will be designed to provide investigators with 24/7 support for the conduct of the study, which will provide an online access to study materials, training module, patient enrollment updates, study newsletter, and relevant literature (e.g. key international literature, trend and dynamics in the outcomes research area, etc.).

## Quality Control

Data quality control will include the following steps:

- EDC edit check and logic check at data entry
- Real time data query reporting and resolution;
- 100% on site monitoring ensuring adequate patient enrollment and consent, proper completetion and documentation of original medical chart, and protocol compliance
- 50% remote source document verification (rSDV) and 10% on-site SDV at each site;

Frequent data query from a specific site will trigger a data audit visit at the site. Randomly selected site auditing will also be performed to ensure proper study conduct.

Lab certification at each site will be obtained prior to enrolling patients.

## Study Duration

The study will be composed of the follow four stages:

1. Study preparation – This includes study material development (CRF, EDC, ICF, etc), site selection and qualification, and EC/IRB approval. The estimated duration is 3 months.
2. Patient enrollment – This period extends from the first to the last patient enrollment (FPI to LPI). The estimated duration is 6 months.
3. Follow up period – This period is 12 months according to the study design.
4. Data Cleaning, analysis and reporting – This period is from the completion of eCRF for all patients to the Clinical Study Report and is estimated to be 3 months.

The total duration of the study is thus estimated to be 24 months (from commencement to CSR).

## Study Structure

The study sponsor is Bristol-Myers Squibb.

The principal investigator of this study is Prof Linong Ji, Co-chairman of CCMR Advisory Board, Director of Endocrinology Department, People’s Hospital of Peking University.

The overall scientific advisory board is the CCMR International Advisory Board [Appendix I – CCMR Execution Plan].

The Study Steering Committee will be composed of national and regional level medical experts recommended by the CCMR Advisory Board.

Study Management will be provided by VitalStrategic Research Institute, the co-organizer of the CCMR.

# ADVERSE EVENT REPORTING

## Investigators’ responsibilities

Investigators must report any Adverse Events arising out of or in connection with the Study to the Regulatory Authority in accordance with applicable laws and regulations; Investigators must report to BMS any Serious Adverse/Adverse Events relating to BMS products within 1 business day of becoming aware. Reports must be made using the CIOMS form and be sent via email to [Safety_China@bms.com] or fax (021-3229 0277). The AE handling schedule is as shown in Table 2 below.

## Definitions of adverse events

**Adverse Event (AE)** is any untoward medical occurrence in a patient or clinical investigation subject administered a pharmaceutical product and which does not necessarily have to have a causal relationship with this treatment.

Therefore, an adverse event can therefore be any unfavourable and unintended sign (including an abnormal laboratory finding, for example), symptom, or disease temporally associated with the use of a medicinal product, whether or not considered related to the medicinal product.

**Serious criteria,** one of the following reactions caused by using the medical product:

1. Results in death;
2. Is life-threatening;
3. Requires inpatient hospitalization or causes prolongation of existing hospitalization;
4. Results in persistent or significant disability/incapacity;
5. Is a congenital anomaly/birth defect;
6. Is an important medical event (defined as a medical event(s) that may not be immediately life-threatening or result in death or hospitalization but, based upon appropriate medical and scientific judgment, may jeopardize the patient/subject or may require intervention (e.g. medical, surgical) to prevent one of the other serious outcomes listed in the definition above.)

Adverse Event Reporting

| **Drugs** | **Description of report** | **Timeframe (calendar day)** | **Transmission** |
| --- | --- | --- | --- |
| Imported drugs within 5 years of first approved in China  (For example Onglyza) | Death | Immediately | Fill out the “Drug Adverse Reaction/Event Report Form”   - **Online** report: [www.adr.gov.cn](http://www.adr.gov.cn)   <Note^[[1]](#footnote-0)^>  Or   - Fax/Email: XXXXXXXX (*ADR Canter where the hospital located)*   <Note^[[2]](#footnote-1)^> |
|  | Serious or unexpected ADR | Within 15 days |  |
|  | Other ADRs | Within 30 days |  |
| Drugs beyond observation period/ imported drugs beyond 5 years of first approved in China  (For example Glucophage, Monopril) | Death | Immediately |  |
|  | Serious or unexpected ADR | within 15 days |  |

IMPORTANT:

1. Death cases shall be reported immediately.
2. Follow-up visits, if any, shall be reported promptly.

# PUBLICATION OF THE RESULTS

All the documents and data related to this study are shared with the CCMR advisory board and the study funder. All publications must be reviewed by the Steering Committee, the study sponsor, the study funder, and produced based on the publication plan. Individual investigators or institutions must obtain the written endorsement from the Steering Committee before they publish any data related to this study.

# COMPLIANCE

No patient private information that is not relevant in accordance to the protocol will be collected for this study. Patients’ identity will be kept anonymized. Investigators will ensure to collect and handle all patient data according to GCP/ICH guidelines, local laws and regulations. All data collected are true, clear and legible. No further manipulation of the data will be conducted and any means that may breach the integrity of data will be prohibited. All data collected are adequate, accurate and according to the study protocol.

The researchers will inform the subject about data collection before the start of the study. The investigator will make awareness of the subject that the data collected in this study can be transferred to the organization or institution in other countries.

Appropriate technical means and management procedures should be adopted to prevent the personal information of the subjects from being accessed or opened to the unauthorized personnel and organizations. No accidental and unlawful damage to the data and accidental loss and change of the data will occur. In the whole study period, the researchers who have the right to look over the personal information of the subjects should keep all information confidential.

# ETHICAL REVIEW

According to the national and local regulations, all the study documents should be filed, formatted and submit to the Ethics Committee (EC) or Institutional Review Board (IRB) for review and approval. No patient medical record can be accessed prior to obtaining an approval from the EC/IRB or other designated authority.

# REFERENCE:

1. Yang WY, Lu JM, Weng JP, et al .Prevalence of Diabetes among Men and Women in China. N Engl J Med. 2010,25 ;362(12):1090-1101
2. Gu, D; Reynolds, K; Wu, X; Chen, J; Duan, X; Reynolds, RF; Whelton, PK; He, J. Inter-ASIA Collaborative Group. Prevalence of the metabolic syndrome and overweight among adults in China. Lancet. 2005; 365:1398–405.
3. Pan, XR; Yang, WY; Li, GW; Liu, J. Prevalence of diabetes and its risk factors in China, 1994. National Diabetes Prevention and Control Co-Operative Group. Diabetes Care. 1997; 20:1664–9.
4. Li, LL; Zhang, YM; Du, JY; Mao, XM; Wu, XH; Ran, XJ; Wang, SG; Bai, L; Li, ND; Sun, XF. Analysis of metabolic features and food composition related to the pathogenesis of type 2 diabetes mellitus in population of Uigurs and Kazaka in Xinjiang. Chin J Endocrinol Metab. 2005; 21:141–2.
5. WHO Global Status Report on Noncommunicable Diseases 2010
6. Kaur J et al, Diabetes and cardiovascular diseases, Am J Ther, 2002 Nov-Dec;9(6):510-5.
7. SM Haffner et al, Mortality from coronary heart disease in subjects with type 2 diabetes and in nondiabetic subjects with and without prior myocardial infarction. *N Engl J Med.* 339: 229–234, 1998
8. Natali A. et al, Coronary atherosclerosis in Type II diabetes: angiographic findings and clinical outcome, Diabetologia, 2000 May; 43(5):632-41.
9. Nationwide Assessment of Cardiovascular Risk Factors: Blood Pressure, Blood Lipid, and Blood Glucose, in Chinese Patients with Type 2 Diabetes CCMR - 3B STUDY (Non-Published)
10. SM Grundy et al, Diabetes and Cardiovascular Disease: A Statement for Healthcare Professionals from the American Heart Association, *Circulation*. 1999; 100:1134-1146.
11. RH Echkel et al, Preventing Cardiovascular Disease and Diabetes, A Call to Action From the American Diabetes Association and the American Heart Association, **Circulation. 2006; 113:2943-2946**
12. Chinese Guidelines for Prevention and Treatment of Diabetes, 2010.
13. IDF Clinical Guidelines Task Force Global Guideline for Type 2 Diabetes: recommendations for standard，comprehensive，and minimal care． Diabet Med，2006，23: 579-593．
14. Pan Chang-yu*，Tian Hui，Liu Guo-liang，LIi Xiu-jun，Fu Zu-zhi，Chen Jia-wei. The Diabcare-Asia（China）Study Group., A survey of diabetes management in Chinese primary healthcare centers Chin J Endocrinol Metab，2004，20：420-424.
15. Wenying Yang, M.D., Juming Lu, M.D., Jianping Weng, M.D., Weiping Jia, M.D., Linong Ji, M.D., et al for the China National Diabetes and Metabolic Disorders Study Group*Gu, D; Reynolds, K; Wu, X; Chen, J; Duan, X; Reynolds, RF; Whelton, PK; He, J. Inter-ASIA Collaborative Group. Prevalence of the metabolic syndrome and overweight among adults in China. Lancet. 2005;365:1398–405.
16. Li GW, Zhang P, Wang J,et al. The long-term effect of lifestyle interventions to prevent diabetes in the China Da Qing Diabetes Prevention Study: a 20-year follow-up study.

Lancet. 2008,371(9626):1783-9.

1. Pan CY，Yang WY，Jia WP，et al． Management of Chinese patients with type 2 diabetes，1998 － 2006: the Diabcare- China surveys． Curr Med Res Opin，2009，25: 39-45．
2. UK Prospective Diabetes Study Group. UK prospective diabetes study 16. Overview of 6 years’ therapy of type II diabetes: a progressive disease. *Diabetes* 1995;44(11):1249–1258.
3. China Guideline For Type 2 Diabetes, Chinese Diabetes Society, 2011
4. Bała, M M, Płaczkiewicz-Jankowska, E, et al the ARETAEUS Study Group, Is newly diagnosed type 2 diabetes treated according to the guidelines? Results of the Polish ARETAEUS1 study. Polish Arch Med Wewn. 2011;121 (1-2): 7-17

**Supplemental eChecklist**

**STROBE Statement—checklist of items that should be included in reports of observational studies**

|  | Item No. | Recommendation | Page  No. | Relevant text from manuscript |
| --- | --- | --- | --- | --- |
| **Title and abstract** | 1 | (*a*) Indicate the study’s design with a commonly used term in the title or the abstract | 1,2 | Title |
|  |  | (*b*) Provide in the abstract an informative and balanced summary of what was done and what was found | 3 | Abstract |
| Introduction | | | |  |
| Background/rationale | 2 | Explain the scientific background and rationale for the investigation being reported | 4 | Introduction |
| Objectives | 3 | State specific objectives, including any prespecified hypotheses | 4 | Introduction |
| Methods | | | |  |
| Study design | 4 | Present key elements of study design early in the paper | 5 | Methods (Study design and Population) |
| Setting | 5 | Describe the setting, locations, and relevant dates, including periods of recruitment, exposure, follow-up, and data collection | 5,6 | Methods (Study design and Population, Study procedures and Data collection) |
| Participants | 6 | (*a*) *Cohort study*—Give the eligibility criteria, and the sources and methods of selection of participants. Describe methods of follow-up  *Case-control study*—Give the eligibility criteria, and the sources and methods of case ascertainment and control selection. Give the rationale for the choice of cases and controls  *Cross-sectional study*—Give the eligibility criteria, and the sources and methods of selection of participants | 5 | Methods (Study design and Population) |
|  |  | (*b*) *Cohort study*—For matched studies, give matching criteria and number of exposed and unexposed  *Case-control study*—For matched studies, give matching criteria and the number of controls per case |  | Not applicable |
| Variables | 7 | Clearly define all outcomes, exposures, predictors, potential confounders, and effect modifiers. Give diagnostic criteria, if applicable | 6,7 | Methods, Supplemental e Protocol |
| Data sources/ measurement | 8* | For each variable of interest, give sources of data and details of methods of assessment (measurement). Describe comparability of assessment methods if there is more than one group | 6 | Methods, Supplemental e Protocol |
| Bias | 9 | Describe any efforts to address potential sources of bias | 6,7 | Methods, Supplemental e Protocol |
| Study size | 10 | Explain how the study size was arrived at | 7 | Methods, Supplemental e Protocol |

Continued on next page

| Quantitative variables | 11 | Explain how quantitative variables were handled in the analyses. If applicable, describe which groupings were chosen and why | 6,7 | Methods, Supplemental e Protocol |
| --- | --- | --- | --- | --- |
| Statistical methods | 12 | (*a*) Describe all statistical methods, including those used to control for confounding | 7 | Methods, Supplemental e Protocol |
|  |  | (*b*) Describe any methods used to examine subgroups and interactions | 7 | Methods, Supplemental e Protocol |
|  |  | (*c*) Explain how missing data were addressed | 7 | Methods, Supplemental e Protocol |
|  |  | (*d*) *Cohort study*—If applicable, explain how loss to follow-up was addressed  *Case-control study*—If applicable, explain how matching of cases and controls was addressed  *Cross-sectional study*—If applicable, describe analytical methods taking account of sampling strategy | 7 | Methods, Supplemental e Protocol |
|  |  | (*e*) Describe any sensitivity analyses | 7 | Methods, Supplemental e Protocol |
| Results | | | | |
| Participants | 13* | (a) Report numbers of individuals at each stage of study—eg numbers potentially eligible, examined for eligibility, confirmed eligible, included in the study, completing follow-up, and analysed | 7,8 | Results, Table 1 and Table 2 |
|  |  | (b) Give reasons for non-participation at each stage | 7,8 | Results, Table 1 and Table 2 |
|  |  | (c) Consider use of a flow diagram | 7,8 | Results, Table 1 and Table 2 |
| Descriptive data | 14* | (a) Give characteristics of study participants (eg demographic, clinical, social) and information on exposures and potential confounders | 7,8 | Results, Table 1 and Table 2, Supplemental Table S1 |
|  |  | (b) Indicate number of participants with missing data for each variable of interest | 7,8 | Results, Table 1 and Table 2 |
|  |  | (c) *Cohort study*—Summarise follow-up time (eg, average and total amount) | 7,8 | Results, Table 1 and Table 2 |
| Outcome data | 15* | *Cohort study*—Report numbers of outcome events or summary measures over time | 7,8 | Results, Table 1 and Table 2 |
|  |  | *Case-control study—*Report numbers in each exposure category, or summary measures of exposure |  | Not applicable |
|  |  | *Cross-sectional study—*Report numbers of outcome events or summary measures |  | Not applicable |
| Main results | 16 | (*a*) Give unadjusted estimates and, if applicable, confounder-adjusted estimates and their precision (eg, 95% confidence interval). Make clear which confounders were adjusted for and why they were included | 7-11 | Results, Table 1, Table 2, and Table 3, Figure 1 |
|  |  | (*b*) Report category boundaries when continuous variables were categorized | 7-11 | Results, Table 1, Table 2, and Table 3, Figure 1 |
|  |  | (*c*) If relevant, consider translating estimates of relative risk into absolute risk for a meaningful time period |  | Not applicable |

Continued on next page

| Other analyses | 17 | Report other analyses done—eg analyses of subgroups and interactions, and sensitivity analyses | 10,11 | Results, Table 2, and Table 3 |
| --- | --- | --- | --- | --- |
| Discussion | | | | |
| Key results | 18 | Summarise key results with reference to study objectives | 12 | Discussion |
| Limitations | 19 | Discuss limitations of the study, taking into account sources of potential bias or imprecision. Discuss both direction and magnitude of any potential bias | 16 | Discussion |
| Interpretation | 20 | Give a cautious overall interpretation of results considering objectives, limitations, multiplicity of analyses, results from similar studies, and other relevant evidence | 12-16 | Discussion |
| Generalisability | 21 | Discuss the generalisability (external validity) of the study results | 16 | Discussion and Conclusion |
| Other information | |  | | |
| Funding | 22 | Give the source of funding and the role of the funders for the present study and, if applicable, for the original study on which the present article is based | 17 | Acknowledgement |

*Give information separately for cases and controls in case-control studies and, if applicable, for exposed and unexposed groups in cohort and cross-sectional studies.

**Note:** An Explanation and Elaboration article discusses each checklist item and gives methodological background and published examples of transparent reporting. The STROBE checklist is best used in conjunction with this article (freely available on the Web sites of PLoS Medicine at http://www.plosmedicine.org/, Annals of Internal Medicine at http://www.annals.org/, and Epidemiology at http://www.epidem.com/). Information on the STROBE Initiative is available at www.strobe-statement.org.

1. [↑](#footnote-ref-0)
2. [↑](#footnote-ref-1)
